# The Tetraspanin CD81 Is a Host Factor for Chikungunya Virus Replication

**DOI:** 10.1128/mbio.00731-22

**Published:** 2022-05-25

**Authors:** Lisa Lasswitz, Francisco J. Zapatero-Belinchón, Rebecca Moeller, Kirsten Hülskötter, Timothée Laurent, Lars-Anders Carlson, Christine Goffinet, Graham Simmons, Wolfgang Baumgärtner, Gisa Gerold

**Affiliations:** a Institute for Experimental Virology, TWINCORE, Centre for Experimental and Clinical Infection Research, a joint venture between the Medical School Hannover and the Helmholtz Centre for Infection Research, Hanover, Germany; b Institute for Biochemistry & Research Center for Emerging Infections and Zoonoses (RIZ), University of Veterinary Medicine Hanover, Hanover, Germany; c Wallenberg Centre for Molecular Medicine (WCMM), Umeå University, Umeå, Sweden; d Department of Clinical Microbiology, Virology, Umeå University, Umeå, Sweden; e Department of Pathology, University of Veterinary Medicine Hanover, Hanover, Germany; f Department of Medical Biochemistry and Biophysics, Umeå University, Umeå, Sweden; g Molecular Infection Medicine Sweden, Umeå University, Umeå, Sweden; h Charité–Universitätsmedizin Berlin, corporate member of Freie Universität Berlin and Humboldt-Universität zu Berlin, Institute of Virology, Berlin, Germany; i Berlin Institute of Health at Charité–Universitätsmedizin Berlin, Berlin, Germany; j Vitalant Research Institute, University of California, San Francisco, California, USA; k Department of Laboratory Medicine, University of California, San Francisco, California, USA; Icahn School of Medicine at Mount Sinai

**Keywords:** chikungunya virus, alphavirus, replication, CD81, tetraspanin, CHIKV, coronavirus, arenavirus

## Abstract

Chikungunya virus (CHIKV) is an arthritogenic reemerging virus replicating in plasma membrane-derived compartments termed “spherules.” Here, we identify the human transmembrane protein CD81 as host factor required for CHIKV replication. Ablation of CD81 results in decreased CHIKV permissiveness, while overexpression enhances infection. CD81 is dispensable for virus uptake but critically required for viral genome replication. Likewise, murine CD81 is crucial for CHIKV permissiveness and is expressed in target cells such as dermal fibroblasts, muscle and liver cells. Whereas related alphaviruses, including Ross River virus (RRV), Semliki Forest virus (SFV), Sindbis virus (SINV) and Venezuelan equine encephalitis virus (VEEV), also depend on CD81 for infection, RNA viruses from other families, such as coronaviruses, replicate independently of CD81. Strikingly, the replication-enhancing function of CD81 is linked to cholesterol binding. These results define a mechanism exploited by alphaviruses to hijack the membrane microdomain-modeling protein CD81 for virus replication through interaction with cholesterol.

## INTRODUCTION

Chikungunya virus (CHIKV) is an arthritogenic reemerging alphavirus which is transmitted by mosquitoes (1, 2). CHIKV was first discovered in Tanzania in the 1950s (3, 4). Since its reemergence in 2004, millions of cases of chikungunya fever have been reported throughout Africa, Asia, and more recently, in Europe and the Americas (5–9). So far, there is no antiviral therapy available, and vaccine candidates are still under investigation (10). The lack of antivirals against CHIKV is reflected by the limited knowledge of host factors, which are critical for CHIKV infection. The cell adhesion molecule matrix remodeling-associated protein 8 (Mxra8) was recently identified as a receptor for CHIKV and is important for pathogenesis (11, 12). However, residual CHIKV infection in the absence of Mxra8 in human cells or mice suggests that additional host factors involved in entry exist (11, 13). Moreover, CHIKV critically depends on the four-and-a-half-LIM domain protein 1 (FHL1) for viral RNA genome replication *in vitro* and *in vivo* (14). Furthermore, the GTPase-activating protein-binding protein 1 (G3BP1) and G3BP2 play a role at the switch from translation to genome amplification of CHIKV (15, 16). Despite knowledge of other factors involved in CHIKV infection, such as components of coat protein complex I and II (COP I and II) machineries (17) or the human autophagy receptor NDP52 (18), there is still an urgent need to identify host factors of CHIKV.

Tetraspanins are evolutionarily conserved proteins consisting of four transmembrane domains, a small and a large extracellular loop, as well as two intracellular tails and a small intracellular loop (19, 20). In humans, 33 tetraspanins have been identified with roles in signal transduction, regulation of cell development, activation, growth, and motility (21–24). Tetraspanins are scaffolding proteins, which interact with one another and various proteins and lipids, thereby forming tetraspanin-enriched microdomains (TEMs) (22–25). Interestingly, several tetraspanins are involved in virus infections at different life cycle steps (26). For instance, CD151 supports human cytomegalovirus (HCMV) penetration into cells and contributes to human papillomavirus type 16 endocytosis (27, 28). Another example is CD9, which accumulates receptors and proteases of coronaviruses at the plasma membrane and thereby facilitates entry (29, 30). Regarding alphaviruses, Sindbis virus (SINV), Semliki Forest virus (SFV) and CHIKV require Tspan 9 for successful viral membrane fusion inside endosomes (31, 32). Lastly, the tetraspanin CD81 is an essential entry factor for hepatitis C virus (HCV), promotes uncoating and budding of influenza A virus, and regulates the reverse transcription of human immunodeficiency virus-1 (HIV-1) (33–36).

A majority of alphaviruses, including CHIKV, replicate their genome in virus infection-induced plasma membrane compartments termed “spherules” (37, 38). These replication compartments contain the four nonstructural proteins (nsp1, nsp2, nsp3, and nsp4) involved in replication. The nsp1 of CHIKV is the only nonstructural protein that has been reported to anchor directly to the plasma membrane and associate with cholesterol-rich microdomains for efficient replication (39, 40). Whether host factors participate in spherule formation remains elusive to date.

In this study, we identify human and murine CD81 as host factors for CHIKV replication. Expression of CD81 enhances CHIKV permissiveness, while a lack of CD81 decreases infection. In addition, related human pathogenic alphaviruses, including SFV, SINV, Ross River virus (RRV) and Venezuelan equine encephalitis virus (VEEV) but not coronaviruses, require CD81 for efficient infection. Finally, we show that CD81 mutants with reduced cholesterol-binding capacity fail to rescue CHIKV infection to wild-type levels. In summary, our results suggest that CD81 is an alphavirus host factor that enhances viral replication by a mechanism that requires cholesterol.

## RESULTS

### CD81 expression enhances chikungunya virus infection of human cells.

**FIG 1:**
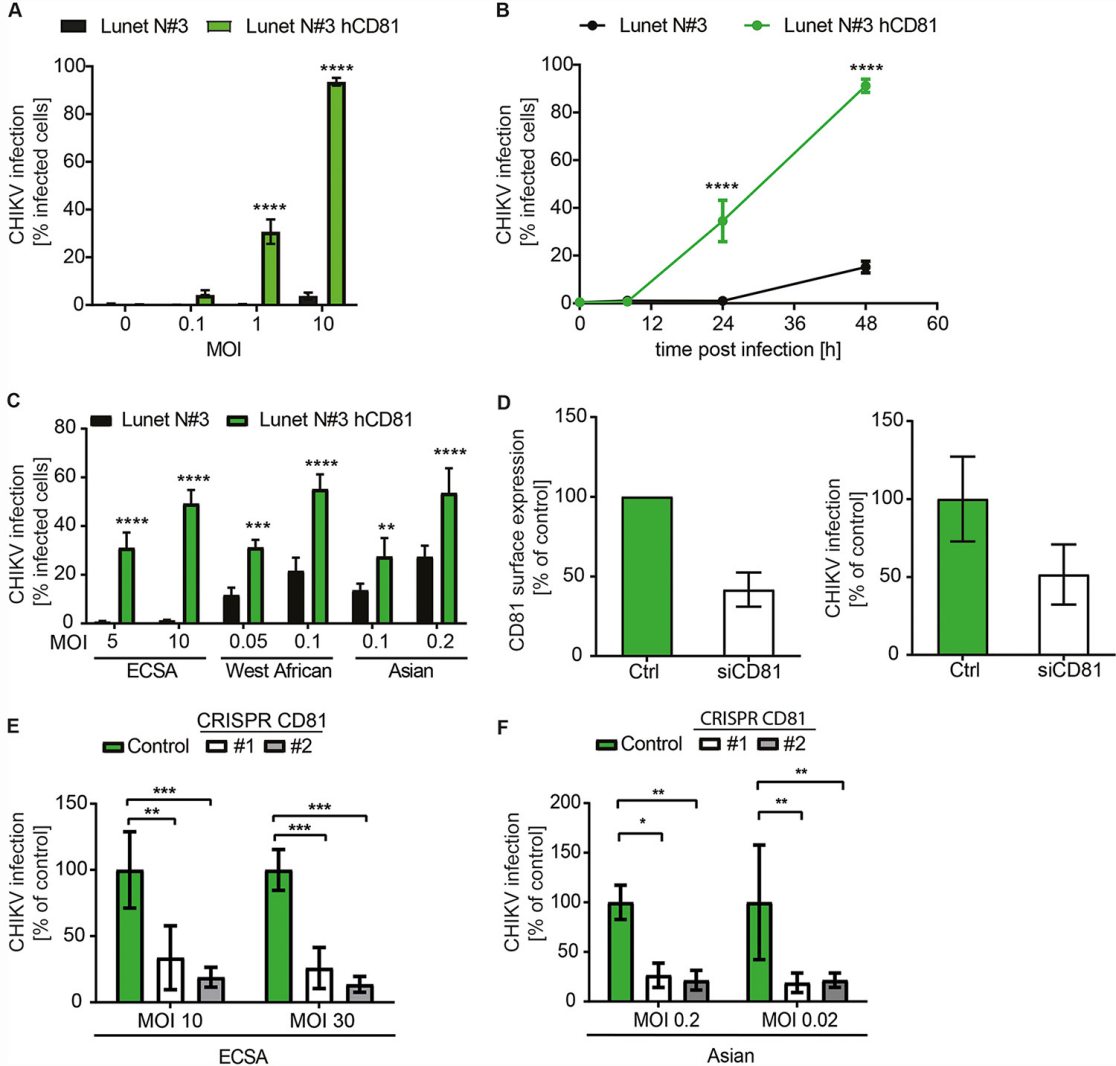
CD81 is a host factor for chikungunya virus. (A) Human hepatoma cells lacking or expressing CD81 were infected with the indicated MOIs of a chikungunya virus (CHIKV) GFP reporter strain (LR2006-OPY1, ECSA genotype). Infectivity was measured as percentage of GFP-positive cells at 24 h postinfection. MOI was based on titration on HEK293T cells. (B) Time course of CHIKV infection (MOI, 1) of indicated cells infected as in panel A. (C) CHIKV strains differentially depend on CD81. Cells expressing or lacking CD81 were infected with CHIKV strains of the ECSA, West African, and Asian genotypes and infectivity measured using fluorescence reporters (GFP, mCherry) at 24 h postinoculation. (D) Huh-7.5 cells silenced with CD81 targeting siRNA or nontargeting siRNA (Ctrl) were infected with the GFP LR2006-OPY1 isolate of CHIKV. Surface expression of CD81 was assessed by antibody staining and flow cytometry (left panel). Infectivity was quantified as GFP signal 24 h postinfection (right panel). (E and F) CD81 gene expression was disrupted by CRISPR/Cas9 in human fibroblasts using a CD81 targeting guide RNA or a nontargeting guide RNA (control). Independent stable cell lines (#1 and #2) were infected with ECSA (E) or Asian (F) CHIKV genotypes, and infection was measured by flow cytometry or luminometry, respectively, 24 h postinfection. All MOIs are based on titration on 293T cells. Data are the mean ± SD of three to four biological replicates, each performed in three technical replicates. Two-way ANOVA with Sidak’s multiple-comparison test (A to C and E). Two-way ANOVA with Dunnett’s multiple-comparison test (F). *, *P* < 0.05; **, *P* < 0.01; ***, *P* < 0.001; ****, *P* < 0.0001.

The tetraspanin CD81 is known to organize plasma membrane microdomains and provide the correct nanoscale organization for membrane bud formation (41). As CHIKV replicates in membrane buds termed spherules, we hypothesized that CD81 might be required for CHIKV infection. To this end, we used the cell culture-derived human hepatoma cell clone Lunet N#3 that lacks endogenous expression of CD81 and cells overexpressing human CD81 (Lunet N#3 hCD81) (42). Cell surface antibody staining confirmed that only Lunet N#3 hCD81 cells display detectable levels of CD81 (Fig. S1A). Loss of CD81 rendered cells almost completely refractory to the LR2006-OPY1 strain (East-Central-South-African [ECSA] genotype) of CHIKV when assessing permissiveness 24 h postinfection. However, upon CD81 expression, cells became highly permissive (31% versus 0.3% and 94% versus 4% compared to parental cells) at a multiplicity of infection (MOI) of 1 and 10, respectively (Fig. 1A). Infection kinetics showed that 48 h postinfection, only 15% of parental Lunet N#3 cells but more than 90% of CD81-expressing cells were infected (Fig. 1B). We confirmed these results in the hepatoma cell line HepG2 in the presence and absence of CD81 (Fig. S1B to D). Interestingly, CD81 dependency was CHIKV strain-specific (Fig. 1C). The globally circulating LR2006-OPY1 strain (ECSA genotype) was highly dependent on CD81 expression, while the CHIKV 37997 strain (West African genotype) and CHIKV 181/25 strain (Asian genotype) showed moderate dependency on CD81 (Fig. 1C). Next, we silenced endogenous CD81 in human hepatoma cells (Huh-7.5) and infected these with the CHIKV ECSA genotype. Transient CD81 silencing with small interfering RNAs (siRNAs) led to a 2-fold reduction of CD81 surface levels and to a concomitant 2-fold reduction in permissiveness to CHIKV (Fig. 1D). Finally, we tested whether CD81 is also a critical host factor in dermal fibroblasts, the primary target of CHIKV (43). To do so, we generated CRISPR/Cas9 CD81 knockout cells and confirmed the lack of CD81 by surface and intracellular staining of CD81 (Fig. S1E). Two independent CD81 knockout cell lines (#1 and #2) showed a 3- to 4-fold (#1) and 5- to 7-fold (#2) reduction in CHIKV permissiveness using an MOI of 10 and 30 of a CHIKV ECSA genotype, respectively (Fig. 1E). We made a similar observation using an Asian CHIKV genotype resulting in a 4- to 5-fold reduction of infectivity in both CD81 knockout cell lines compared to control cells (Fig. 1F). Altogether, our results suggest that human CD81 is a CHIKV host factor in human cells.

10.1128/mbio.00731-22.1 FIG S1 (A) Cell surface staining of hCD81 on Lunet N#3 (upper panel) and Lunet N#3 hCD81 cells (lower panel) using anti-hCD81 antibody (JS-81-APC) or an isotype control (IgG1, κ). Unstained cells served as the negative control. (B) HepG2 and HepG2 hCD81 cells were surface stained as in panel A and analyzed by flow cytometry. (C) HepG2 cells lacking or expressing hCD81 were infected with the indicated MOIs of a GFP reporter CHIKV strain (LR2006-OPY1). Infectivity was measured as the percentage of GFP-positive cells at 24 h postinfection. (D) Time course of CHIKV infection (MOI, 1) of indicated cells infected as in panel C. (E) CD81 gene expression was disrupted by CRISPR/Cas9 in human fibroblasts using a CD81 targeting guide RNA or a nontargeting guide RNA (control). CD81 surface and intracellular staining (as in panel A) of control cells and two independent stable cell lines (#1 and #2) was quantified by flow cytometry. Unstained cells served as negative control. Data are the mean ± SD of three biological replicates, each performed in three technical replicates (C and D). Two-way ANOVA with Sidak’s multiple-comparison test (C and D); *, *P* < 0.05; **, *P* < 0.01; ****, *P* < 0.0001. Download FIG S1, TIF file, 1.4 MB. Copyright © 2022 Lasswitz et al. 2022 Lasswitz et al. https://creativecommons.org/licenses/by/4.0/ This content is distributed under the terms of the Creative Commons Attribution 4.0 International license.

### Murine CD81 is a host factor for chikungunya virus and is expressed in virus target tissues.

**FIG 2:**
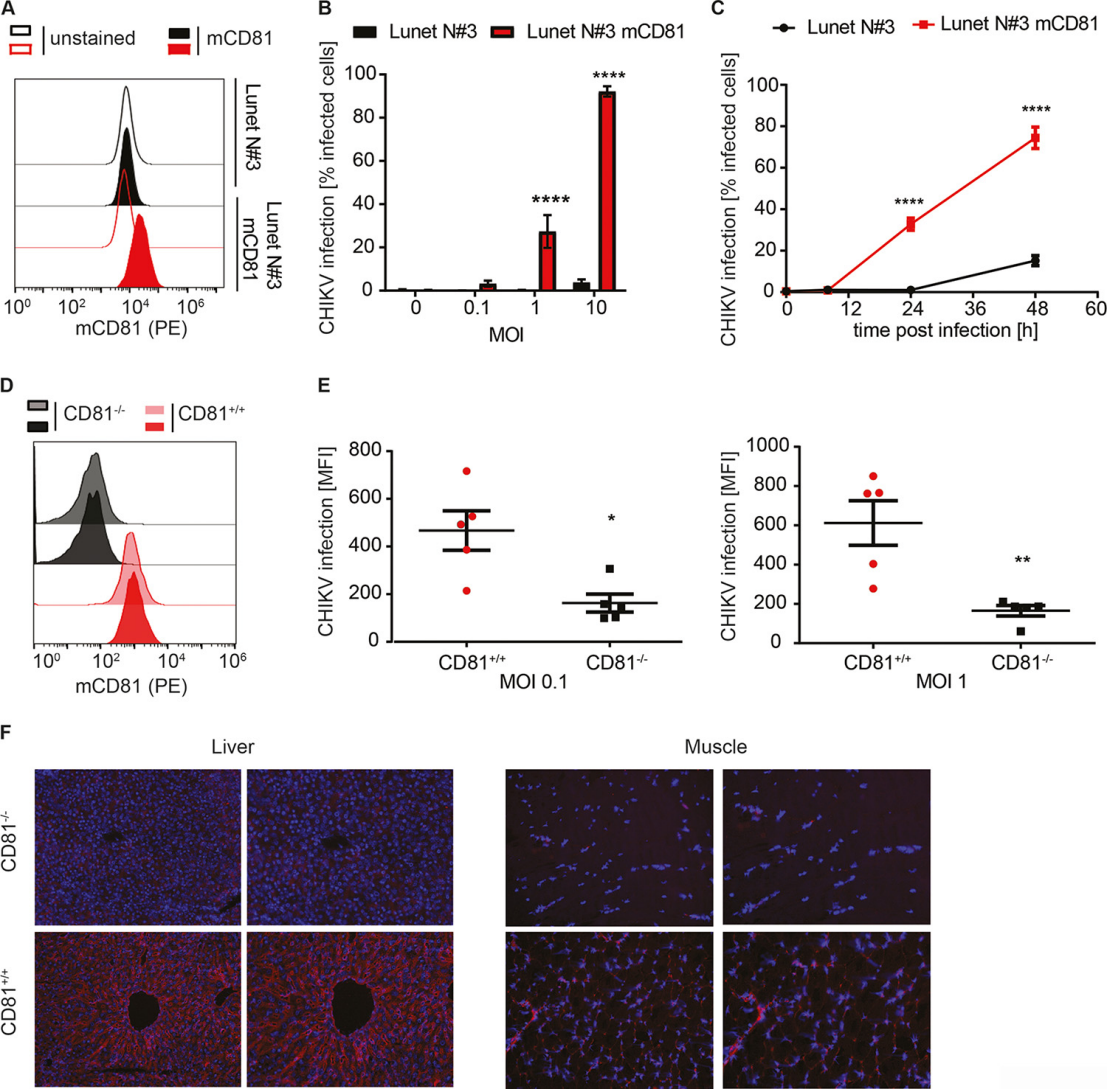
Murine CD81 is a host factor for CHIKV and is expressed in susceptible tissues. (A) Ectopic expression of murine CD81 (mCD81) in human hepatoma cells (red histograms) assessed by anti-mouse CD81 antibody (EAT-2-PE) staining (filled histograms) and flow cytometry. Unstained cells (open histograms) and cells lacking mCD81 expression (black histograms) served as negative controls. (B) Lunet N#3 and Lunet N#3 mCD81 cells were infected with the indicated MOIs of a CHIKV GFP reporter strain (LR2006-OPY1, ECSA genotype). Susceptibility was measured as the percentage of GFP-positive cells at 24 h postinfection by flow cytometry. (C) Time course of CHIKV infection (MOI, 1) of indicated cells infected as in panel B. (D) Flow cytometry analysis of primary mouse fibroblasts isolated from wild-type or CD81 knockout mice stained with an anti-mouse CD81 antibody (EAT-2-PE). Displayed are representative histograms from cells isolated from two wild-type or two knockout (CD81−/−) mice. (E) Primary mouse fibroblasts isolated from five wild-type and five knockout mice were infected with the indicated MOIs of a CHIKV GFP reporter strain (LR2006-OPY1). Infectivity was measured by flow cytometry 24 h postinfection. MOI are based on titration on 293T cells. (F) Immunostaining of mCD81 (EAT-2, red) and nuclei (bisbenzimide, blue) in liver and muscle tissue of wild-type or CD81 knockout mice (CD81−/−). The images on the right display a 4×-digital zoom of the respective image on the left. All MOIs are based on titration on 293T cells. Data are shown as the mean ± SD of three biological replicates, each performed in three technical replicates (B and C). Two-way ANOVA with Sidak’s multiple-comparison test (B and C). The data in panel E are the mean ± SEM with *n* = 5 for each group (wildtype and CD81 knockout) and two-tailed Student’s *t* test. *, *P* < 0.05; **, *P* < 0.01; ****, *P* < 0.0001.

To investigate whether CD81 is a human-specific CHIKV host factor, we evaluated whether overexpression of murine CD81 (mCD81) also enhances infection of CHIKV LR2006-OPY1 in Lunet N#3 cells (Lunet N#3 mCD81) (42). Flow cytometry analysis confirmed expression of murine CD81 (Fig. 2A), and this expression increased CHIKV infection levels (Fig. 2B and C). Twenty-four hours postinfection, we detected a significant increase (27% versus 0.32% and 92% versus 3.7%) in CHIKV permissiveness of Lunet N#3 mCD81 compared to Lunet N#3 cells at MOIs of 1 and 10, respectively (Fig. 2B). At 48 h postinfection and an MOI of 1, CHIKV infected 15% and 75% of cells in the absence and presence of mCD81, respectively (Fig. 2C). We made similar observations in HepG2 cells expressing or lacking mCD81 (Fig. S2A to C). Next, we tested whether CD81 expression also affected CHIKV infection of primary murine cells. To this end, we isolated and cultured dermal mouse fibroblasts from five wild-type and five CD81 knockout mice as described previously (44, 45). Three days postisolation, cultured cells showed fibroblast-like morphology (Fig. S2D). We confirmed CD81 expression in wild-type and lack of CD81 expression in CD81 knockout fibroblasts by surface staining and flow cytometry (Fig. 2D and Fig. S2E). Next, we infected wild-type and knockout cells with CHIKV (LR2006-OPY1 strain) at an MOI of 0.1 and 1. The absence of CD81 led to a 2.9-fold (MOI, 0.1) or 3.7-fold (MOI, 1) decrease of CHIKV infection in fibroblasts from CD81 knockout mice compared to fibroblasts isolated from wild-type mice (Fig. 2E). Lastly, we analyzed CD81 expression in other tissues targeted by CHIKV. Immunolabeling of murine CD81 in liver and muscle tissue showed expression of the protein in wild-type mice but not in CD81 knockout mice (Fig. 2F). These data show that murine CD81 enhances CHIKV infection in primary dermal fibroblasts and is present in tissues relevant to CHIKV pathogenesis.

10.1128/mbio.00731-22.2 FIG S2 (A) Flow cytometry analysis of HepG2 cells lacking or expressing the murine CD81 (mCD81) stained with an anti-mouse CD81 antibody (EAT-2-PE). Unstained cells served as the negative control. (B) HepG2 and HepG2 mCD81 were infected with the indicated MOIs of a CHIKV GFP reporter strain (LR2006-OPY1). Infectivity was measured as the percentage of GFP-positive cells at 24 h postinfection by flow cytometry. (C) Infection kinetic of CHIKV infection (MOI, 1) of the indicated cells infected as in panel B. (D) Representative bright-field images of primary dermal mouse fibroblast isolated from two wild-type (wt) or two CD81 knockout mice (CD81−/−) after 3 days of culture. Images were captured at ×10 magnification using a light microscope. (E) Flow cytometry analysis of primary dermal mouse fibroblasts from five wild-type and five CD81−/− mice stained with an anti-mouse CD81 antibody (EAT-2-PE). Representative histograms from unstained cells isolated from one mouse of each group are shown on the top and used as the negative control. Data are the mean ± SD of three biological replicates, each performed in three technical replicates (B and C). Two-way ANOVA with Sidak’s multiple-comparison test (B and C); ****, *P* < 0.0001. Download FIG S2, TIF file, 2.7 MB. Copyright © 2022 Lasswitz et al. 2022 Lasswitz et al. https://creativecommons.org/licenses/by/4.0/ This content is distributed under the terms of the Creative Commons Attribution 4.0 International license.

### Diverse human pathogenic alphaviruses require CD81 for efficient infection.

**FIG 3:**
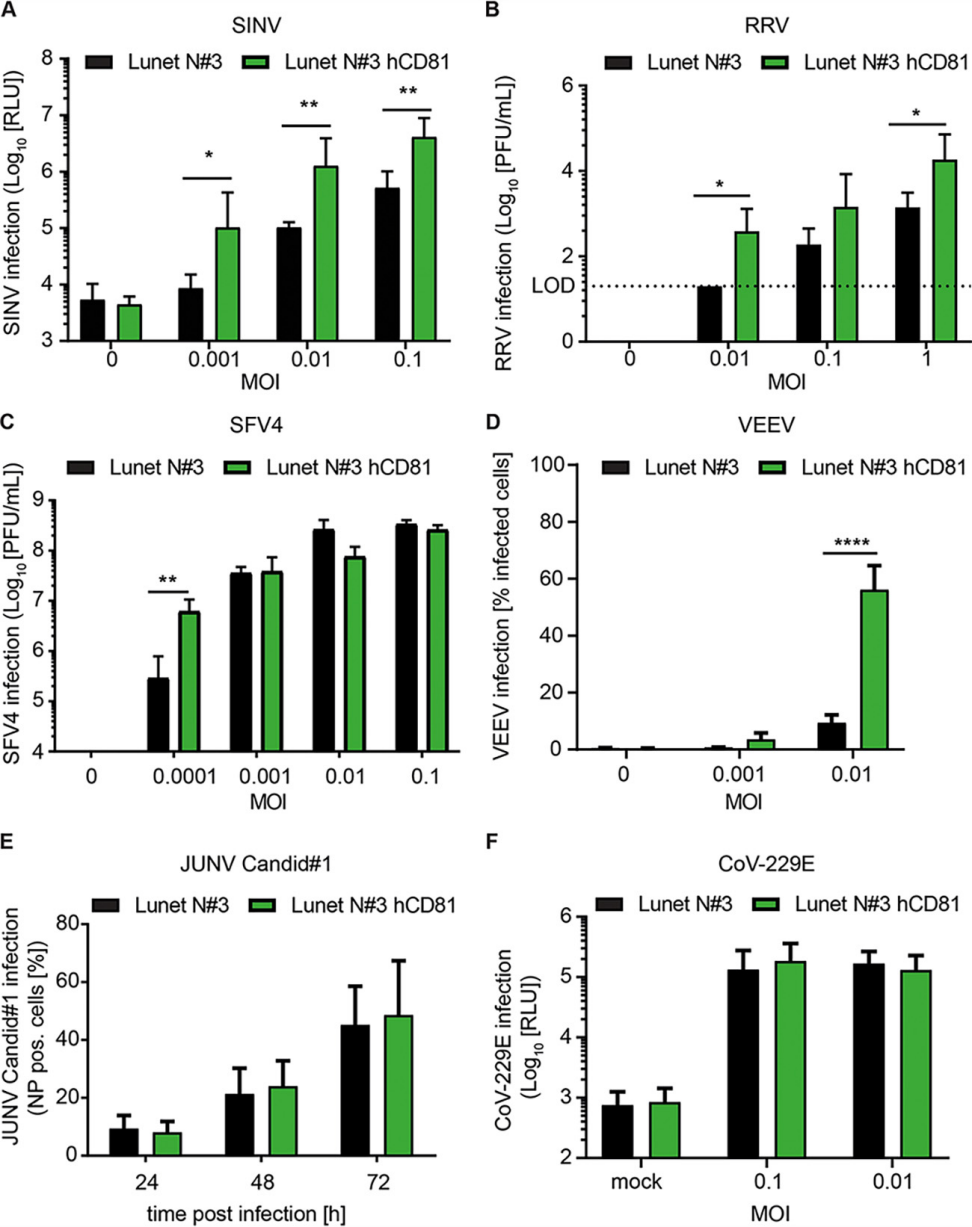
CD81 confers susceptibility to alphaviruses but not to other tested RNA viruses. (A to D) Human hepatoma cells lacking or expressing human CD81 (hCD81) were infected with Sindbis virus (SINV) (A), Ross River virus (RRV) (B), Semliki Forest virus (SFV4) (C), and Venezuelan equine encephalitis virus (VEEV, strain TC-83-GFP) (D) with the indicated MOIs (based on titration on BHK-21 cells). (A) SINV infection was quantified by luciferase activity 24 h after infection. (B and C) Infection with RRV and SFV4 was analyzed by PFU. Cells lacking or expressing CD81 were infected with the indicated MOIs for 2 h prior to inoculum removal. Cell culture supernatants were collected 24 h after infection and titrated on BHK-21 cells by plaque assay. After 72 h (RRV) or 48 h (SFV4), posttitration PFU were determined. (D) Infection of VEEV was assessed by flow cytometry 24 h postinfection. (E) Human hepatoma cells lacking or expressing hCD81 were infected with Junin Candid#1 for the indicated times using an MOI of 1 (based on titration on Vero cells). Infection was quantified by flow cytometry using an anti-NP antibody (clone MA03-BE06). (F) The indicated cells were infected with human coronavirus 229E (CoV-229E). Infection was measured 48 h postinfection by luciferase activity. Data are the mean + SD of three biological replicates, each performed in three technical replicates (A and D), duplicates (E), or quadruplicates (F). Two-way ANOVA with Sidak’s multiple-comparison test (A to F); *, *P* < 0.05; **, *P* < 0.01; ****, *P* < 0.0001.

CHIKV is a member of the family *Togaviridae*, genus *Alphavirus*, which comprises important human and veterinary pathogens. While CHIKV, RRV, and SFV belong to the SF complex, SINV belongs to the Western equine encephalitis (WEE) complex and VEEV to the VEE complex (46). In order to elucidate if CD81 is a broad alphavirus host factor, we tested the CD81 dependency of SINV, RRV, SFV, and VEEV. Expression of CD81 in Lunet N#3 cells resulted in a 10-fold increase in permissiveness to the Old World alphavirus SINV for all tested MOIs (Fig. 3A). RRV permissiveness increased by up to 1.5 log when CD81 was expressed (Fig. 3B), and SFV4 showed a 22-fold increase in viral titers upon CD81 expression at the lowest MOI tested (0.0001 based on titration on BHK-21 cells). However, we did not detect a difference in viral titers of SFV4 between cells lacking or expressing CD81 using higher MOIs (0.001 to 0.1) (Fig. 3C). Lastly, CD81 expression enhanced infection with the New World alphavirus VEEV by 6-fold compared to parental cells (Fig. 3D). In contrast, infection of other enveloped RNA viruses such as the arenavirus Junin virus (JUNV; Candid #1) (Fig. 3E) or human coronavirus 229E (CoV-229E) did not depend on CD81 at any of the MOIs tested (Fig. 3F). Taken together, our results suggest that CD81 is a pan-alphavirus host factor.

### CD81 is dispensable for chikungunya virus entry but critical for viral genome replication.

**FIG 4:**
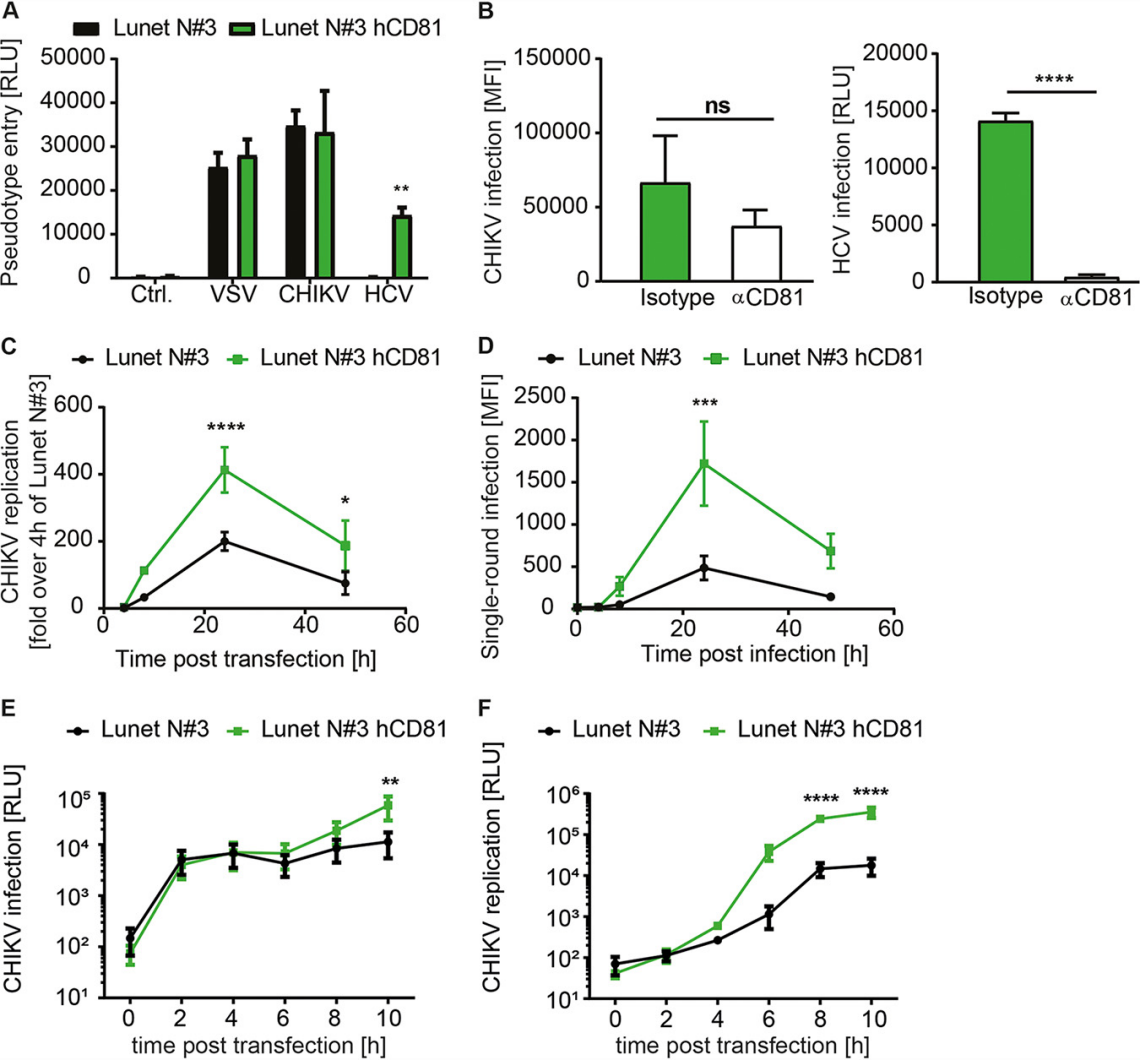
CD81 is dispensable for CHIKV entry but critical for genome replication. (A) Entry of lentiviral particles encoding for firefly luciferase and pseudotyped with glycoproteins of either CHIKV, HCV, or VSV or without envelope protein (Ctrl.). Luciferase activity was measured 72 h postransduction in lysates from indicated cells. (B) Lunet N#3 hCD81 cells were incubated with an anti-CD81 monoclonal antibody (JS-81) or isotype control for 30 min, followed by infection with a CHIKV GFP reporter strain (LR2006-OPY1, ECSA genotype) (left panel) or HCV genotype 2a reporter virus as control (right panel). Infection was measured by flow cytometry 24 h postinfection for CHIKV or luminometrically for HCV 48 h postinfection. (C) Human hepatoma cells lacking or expressing human CD81 (hCD81) were transfected with CHIKV replicon RNA (LR2006-OPY1) containing the nonstructural proteins and a GFP reporter. Replication was analyzed by flow cytometry at the indicated time points posttransfection. Results are normalized to 4-h values of Lunet N#3. (D) Infection of indicated cells with single-round infection particles (LR2006-OPY1). Infectivity was measured as in panel C. (E) CHIKV infection of the indicated cells with luciferase reporter strain (Asian genotype). Permissiveness was measured at the indicated time points postinfection by luciferase assay. (F) Full-length RNA transfection with luciferase reporter strain (Asian genotype). Infection was measured at the indicated time points posttransfection by luciferase assay. Data are the mean + SD (A and B)) or mean ± SD (C to F) of three to four biological replicates, each performed in two to three technical replicates. Two-way ANOVA with Sidak’s multiple-comparison test (A and C to F). Two-tailed Student’s *t* test (B). *, *P* < 0.05; **, *P* < 0.01; ***, *P* < 0.001; ****, *P* < 0.0001.

Previous studies have linked CD81 to cell entry of viruses, bacteria, and parasites (33, 35, 47, 48). CD81 is primarily expressed at the plasma membrane, where it coordinates TEMs (23, 24, 49–51). Hence, we investigated if CD81 promoted cell entry of CHIKV particles. To study virus entry, we generated HIV-based lentiviral particles bearing CHIKV glycoproteins. Lentiviral particles harboring the glycoproteins of either vesicular stomatitis virus (VSV-G) or HCV served as positive controls. Lentiviral particles without any viral envelope glycoprotein served as the negative control (Ctrl). As expected, Ctrl particles failed to infect human hepatoma cells, while particles decorated with VSV-G equally transduced hepatoma cells with and without CD81 (Fig. 4A). Lentiviral particles with HCV envelope glycoproteins only transduced CD81-expressing cells, confirming that CD81 is crucial for HCV entry (33, 34, 52). In contrast, particles harboring CHIKV glycoproteins transduced cells independently of CD81 expression, suggesting that CD81 is dispensable for early steps of CHIKV infection. To confirm this notion by an orthogonal method, we preincubated cells with a human CD81 (hCD81) ectodomain-specific monoclonal antibody (clone JS-81) to block CD81 ectodomain-dependent functions on the cell surface of Lunet N#3 hCD81 cells. After preincubation with anti-CD81 or an isotype control antibody, we infected cells with CHIKV (LR2006-OPY1 strain) or cell-culture-derived HCV (genotype 2a; JcR2a). While CD81 blockage abrogated HCV entry as expected, it had no significant influence on CHIKV infection (Fig. 4B). Together, these data indicate that CD81 is not an entry factor for CHIKV.

Most alphaviruses, including CHIKV, replicate their RNA genomes in plasma membrane-derived compartments (37, 38). Therefore, we investigated whether CHIKV depends on CD81 for viral replication. To do so, we used a CHIKV replicon system that expresses the four nonstructural proteins and hence replicates its genome but is incapable of packaging new virions or spreading in culture. Upon transfection of *in vitro*-transcribed viral replicon RNA, we observed a significant 2-fold increase of viral genome replication when CD81 was present (Fig. 4C). To confirm the CD81-dependent enhancement of viral genome replication, we used virus replicon particles (VRPs), which contain a genome encoding the nonstructural proteins and an enhanced green fluorescent protein (EGFP) reporter. These VRPs enter cells and replicate but cannot package new particles, resulting in a single-round infection (53). Expression of CD81 increased the EGFP signal 24 h post-CHIKV VRP infection by 3- to 4-fold (Fig. 4D). Thus, in two independent assays, CD81 enhanced CHIKV RNA replication.

Alphavirus replication is a two-step process involving early amplification of genomic viral RNA and late amplification of a subgenomic RNA that encodes for viral structural proteins (1). Therefore, the replicon and the single-round infection assay only partially mimic alphavirus genome replication. To assess the role of CD81 in full-length genome replication, we infected cells with a luciferase reporter virus (181/25 strain) and monitored infection kinetics between 2 h and 10 h. While we observed similar levels of luciferase activity at early time points postinfection (2 h and 4 h) in cells expressing CD81 or not, reporter activity increased up to 7-fold in the presence of CD81 at 10 h postinfection (Fig. 4E). This suggests that similar numbers of virus particles entered the cells, resulting in similar early translation levels of incoming genomes, but genome replication was favored in the presence of CD81. To confirm this observation, we next transfected cells with full-length viral RNA of the same 181/25 CHIKV strain. We again observed comparable levels of luciferase activity at 2 h and 4 h posttransfection in cells with and without CD81, indicating similar delivery efficiency of viral genomic RNA into both cell lines (Fig. 4F). However, CD81-expressing cells showed an order of magnitude higher luciferase activity levels at later time points (6 h, 8 h, and 10 h) (Fig. 4F). Collectively, these data suggest that CD81 enhances CHIKV replication but is dispensable for virus entry.

### CD81 colocalizes with CHIKV dsRNA at virus replication sites.

**FIG 5:**
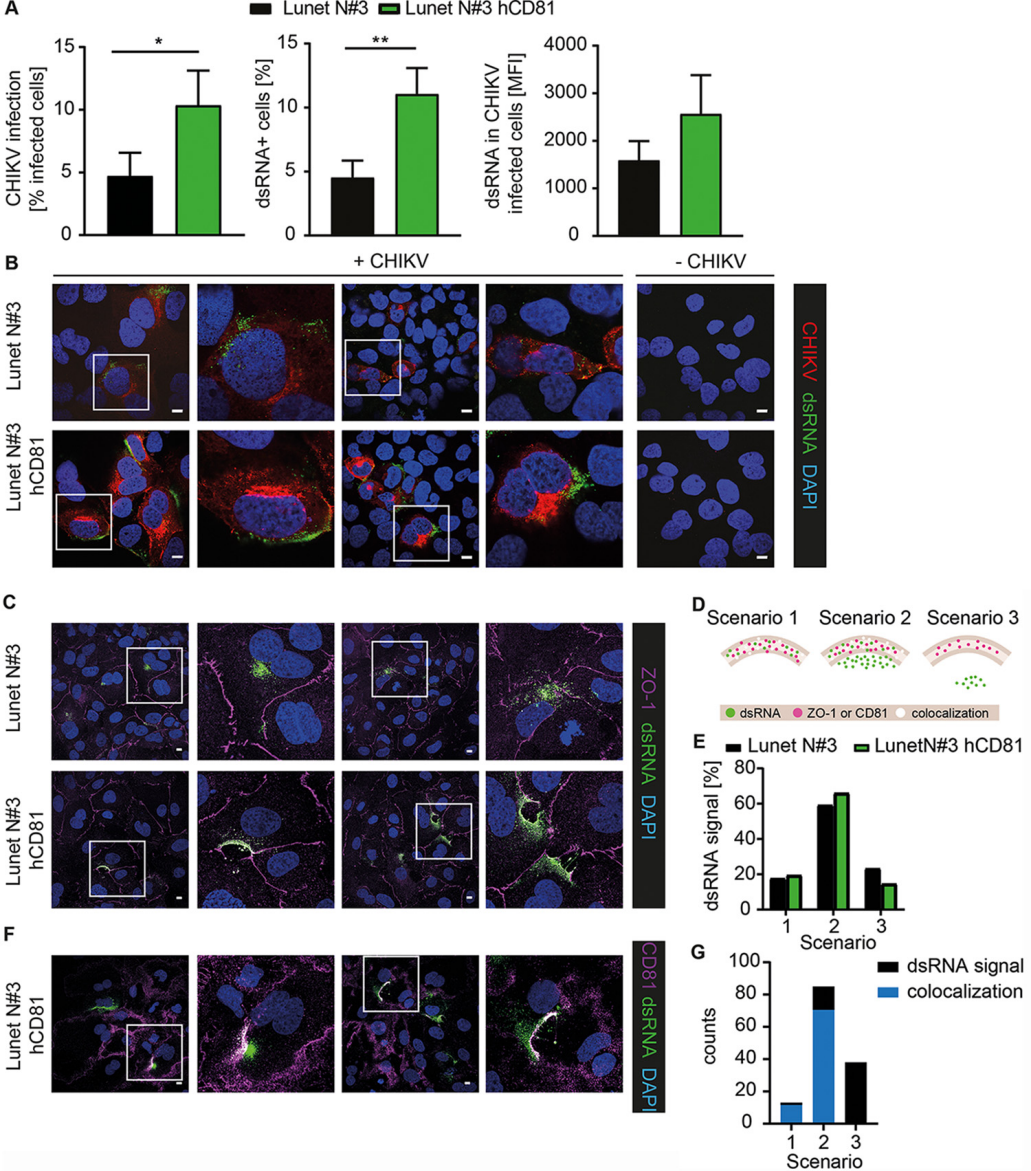
CD81 colocalizes with dsRNA at the plasma membrane. (A and B) Human hepatoma cells lacking or expressing hCD81 were infected with E2-mCherry reporter CHIKV (Asian genotype) for 9 h (MOI, 50; based on titration on 293T cells). Cells were stained with anti-dsRNA antibody (green) and analyzed by flow cytometry (A) or confocal-microscopy (B). (C) Lunet N#3 and Lunet N#3 hCD81 cells were infected as in panels A and B. Cells were stained with anti-dsRNA antibody (green), anti-ZO-1 antibody (purple), and DAPI (blue). (D) Schematic representation of scenarios used for quantification of images from panels C and F. (E) Quantification of at least 50 infected cells stained as in panel C and shown as dsRNA signal found in scenarios 1 to 3 as depicted in panel D. (F) Lunet N#3 hCD81 cells were infected as in panels A to C. Cells were stained with anti-dsRNA antibody (green), anti-CD81 antibody (purple), and DAPI (blue). (G) Quantification of 136 infected cells stained as in panel F and represented as the number of cells with dsRNA signal in scenarios 1 to 3 (black bars) and the number of CD81-dsRNA colocalization events (blue bars) for the respective scenarios. (A) Data represent the mean + SD of three biological replicates performed in triplicates. Statistical analysis was based on two-tailed Student’s *t* test. *, *P* < 0.05; **, *P* < 0.01. (B, C, and F) Representative images are shown. A 2× digital zoom of the white-framed insets is displayed to the right of each respective image. Scale bars, 10 μm.

After demonstrating that CD81 is involved in CHIKV genome replication, we determined the production of double-stranded RNA (dsRNA) intermediates, a marker for active replication (38), 9 h postinfection using an E2-mCherry CHIKV (Asian genotype). E1/E2 glycoproteins are posttranslationally modified in and transit through the endoplasmic reticulum (ER)-Golgi secretory pathway, resulting in a prominent mCherry signal at the Golgi and labeling of infected cells with mCherry. Flow cytometry analysis revealed a 2-fold increase in CHIKV infectivity (mCherry positive cells) and a concomitant 2.5-fold increase in dsRNA-positive cells upon CD81 expression (Fig. 5A, left and middle panels). In addition, the mean fluorescence intensity of dsRNA in infected cells was 1.6-fold higher in cells expressing CD81 than in those lacking CD81 (Fig. 5A, right panel). Confocal-microscopy of infected cells, stained with dsRNA antibody, confirmed the flow cytometric observation that CD81 expression leads to a stronger dsRNA signal (Fig. 5B). In addition, we observed a prominent dsRNA signal near the plasma membrane, where replication compartments are located (37, 38) (Fig. 5B). In contrast, cells lacking CD81 expression showed a more diffuse staining of dsRNA. To further determine the subcellular localization of dsRNA replication intermediates, we stained infected cells lacking or expressing CD81 with antibodies against dsRNA and concomitantly against the plasma membrane marker zonula occludens 1 (ZO-1). The dsRNA signal in cells expressing CD81 showed a distinct signal at the plasma membrane overlapping with the ZO-1 staining (Fig. 5C). In contrast, the dsRNA signal in cells lacking CD81 was rather dispersed. In order to quantify the dsRNA localization, at least 50 infected cells per cell line were analyzed using the three different localization scenarios represented in Fig. 5D. Scenario 1 refers to a signal of dsRNA exclusively present at the plasma membrane (complete overlap with ZO-1), whereas in scenario 2 a dsRNA signal localized to the plasma membrane (overlap with ZO-1) and to the cytoplasm (close to ZO-1). Scenario 3 represents a dsRNA signal only present in the cytoplasm without any overlap with ZO-1. As the ZO-1 staining is quite narrow compared to the broad dsRNA signal, we detected only 17% and 19% of infection events for scenario 1 in Lunet N#3 cells and Lunet N#3 hCD81 cells, respectively (Fig. 5E). The majority of infection events fell into the category of scenario 2 with a broad dsRNA signal overlapping the ZO-1 staining. We detected slightly more infection events for scenario 2 in cells expressing CD81 (66% versus 59% in cells lacking CD81) and slightly less dsRNA signal in the cytoplasm when CD81 was present (14% versus 23% in cells lacking CD81, scenario 3). Next, we analyzed whether CD81 and dsRNA colocalize at the plasma membrane. To this end, we stained Lunet N#3 hCD81 cells with anti-dsRNA and anti-CD81 antibodies 9 h postinfection (Fig. 5F). Confocal images revealed colocalization of CD81 and dsRNA near the plasma membrane (white signal), where spherules are formed. We quantified the dsRNA signal in relation to the CD81 signal using the three scenarios, similar to what was described for the ZO-1 staining. In addition, we determined the colocalization events between CD81 and dsRNA for the respective scenarios (Fig. 5G). Comparable to the dsRNA and ZO-1 analysis, scenario 2 was the most frequent for dsRNA and CD81 overlap, and we detected colocalization in 71 out of 85 of all scenario 2 events. Out of 136 total events, 13 represented scenario 1, i.e., full overlap of dsRNA and CD81 signal subcellular localization, and concomitantly, nearly the entire dsRNA signal colocalized with the CD81 signal. We did not detect any colocalization for scenario 3 as expected per definition. Together, these data suggest that CD81 promotes CHIKV replication at and in proximity to the plasma membrane.

### CD81 cholesterol binding is required for the enhancement of CHIKV infection.

**FIG 6:**
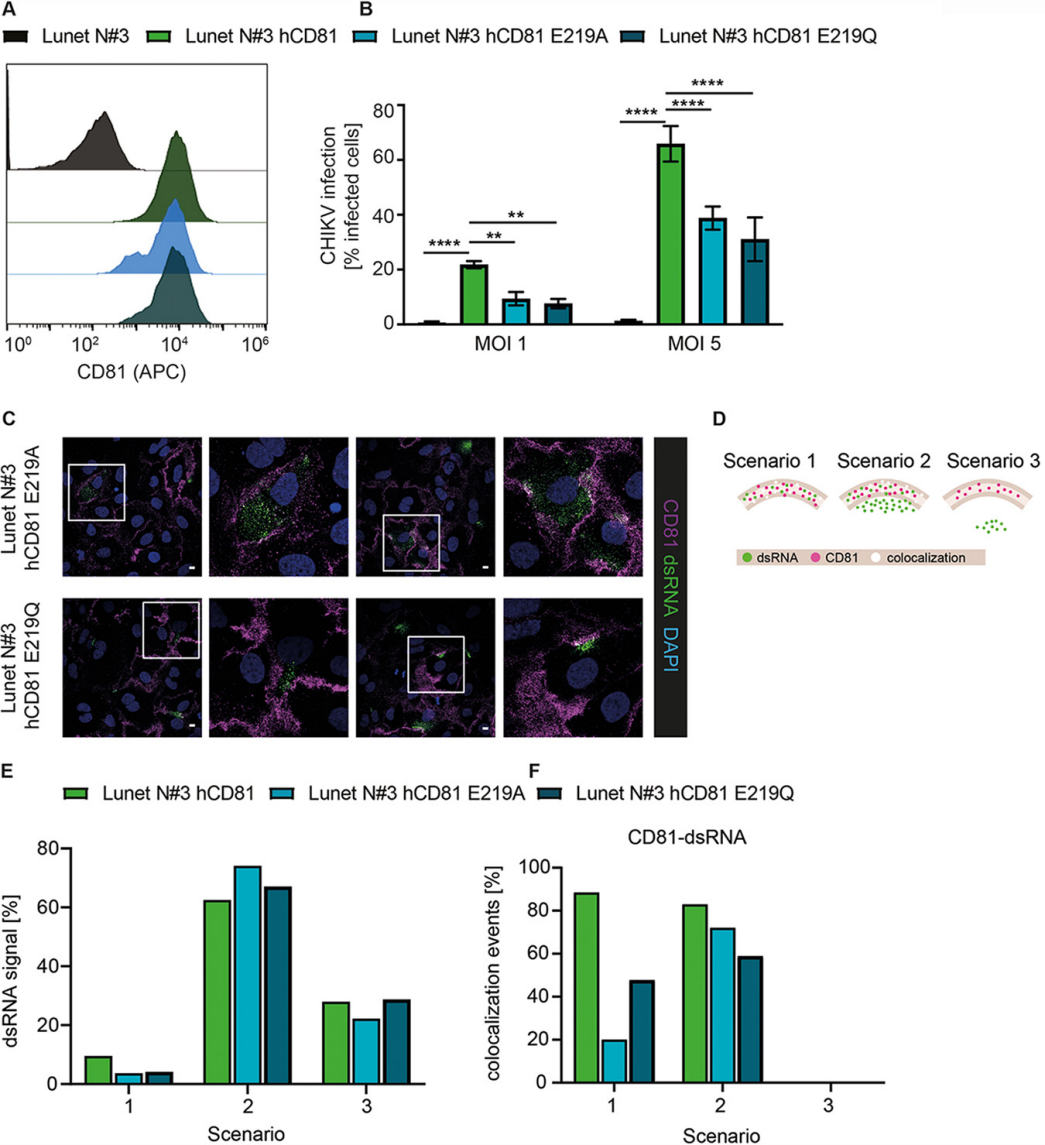
CD81 mutants with reduced cholesterol-binding capacity show reduced CHIKV host factor function. (A) CD81 surface staining of cells without hCD81, with hCD81 wild type, and with hCD81 E219A and E219Q cholesterol-binding mutants (anti-hCD81 antibody, JS-81-APC). (B) The indicated cells were infected with an MOI of 1 and 5 of a CHIKV GFP reporter strain (ECSA genotype; titration based on 293T cells). Infectivity was measured at 24 h postinfection by flow cytometry. Data represent the mean ± SD of three biological replicates, each performed in three technical replicates. Two-way ANOVA with Dunnett’s multiple-comparison test. **, *P* < 0.01; ****, *P* < 0.0001. (C) Human hepatoma cells expressing hCD81 E219A or hCD81 E219Q were infected for 9 h with E2-mCherry reporter CHIKV (Asian genotype; MOI, 50; based on titration on 293T cells) as in Fig. 5F. Cells were stained with anti-dsRNA antibody (green), anti-CD81 antibody (purple), and DAPI (blue). Cells were analyzed by confocal microscopy, and representative images are shown. A 2× digital zoom of the white insets is displayed to the right of each respective image. Scale bars, 10 μm. (D) Schematic representation of scenarios 1 to 3 used for quantification of images from panel C. (E and F) Quantification of at least 68 infected cells stained as in panel C represented as dsRNA signal found in scenarios 1 to 3 (E) and CD81-dsRNA colocalization events for each scenario (F). Representative images for Lunet N#3 hCD81 are shown as in Fig. 5F.

The observation that CD81 was essential for permissiveness of cells from two different species and for alphaviruses from three distinct complexes (VEE complex, WEE complex, and SF complex), led us to hypothesize that CD81 supports a universal mechanism of replication. Cholesterol is a ubiquitous component of biological membranes and critical for regulating membrane fluidity and shape. During alphavirus replication, cholesterol is critically required, and the nsP1 protein of CHIKV, which is part of the replication complex, associates with cholesterol-rich microdomains (39). As CD81 directly binds cholesterol within the membrane, we tested if cholesterol-binding mutants of CD81 (i.e., E219A and E219Q) affect the protein’s function as a host factor (54). To this end, we used Lunet N#3 cells expressing either wild-type hCD81 or hCD81 with the E219A and E219Q point mutations (55) and confirmed that surface expression of all three CD81 variants was comparable (Fig. 6A). As expected, Lunet N#3 cells were poorly permissive to CHIKV (LR2006-OPY1 strain) infection, and expression of wild-type CD81 resulted in a marked increase in CHIKV infection (Fig. 6B). Strikingly, CHIKV infection was significantly decreased by a factor of 2.1 to 2.7 in cells expressing E219A or E219Q mutants, respectively (Fig. 6B). This is in line with previous reports that cholesterol binding of the E219A and E219Q mutants of CD81 is also only moderately reduced by a factor of 2 (54). This may explain the gradual effect of the mutations on CD81 host factor function. Next, we asked whether the localization of dsRNA signal differed between wild-type CD81 and the cholesterol-binding mutants. To this end, we stained infected Lunet cells expressing either CD81 E219A or CD81 E219Q with dsRNA and CD81 antibodies (Fig. 6C) and quantified the dsRNA localization using the three different localization scenarios as described above (Fig. 6D). In comparison to wild-type CD81, both cholesterol-binding mutants had 60% less frequent dsRNA signal in scenario 1, i.e., overlap of dsRNA and CD81 was reduced (Fig. 6E). Interestingly, we observed not only a reduced number of infection events in scenario 1 for both cholesterol-binding mutants, but also a reduction of CD81-dsRNA colocalization events (77% reduction for CD81 E219A and 46% for CD81 E219Q compared to wild-type CD81; Fig. 6F). The dsRNA signal for both cholesterol mutants was more diffuse, correlating with a slight increase in dsRNA signal in scenario 2 in comparison to wild-type CD81 (Fig. 6C and D). Concomitantly, the cholesterol mutants of CD81 also displayed fewer CD81-dsRNA colocalization events in scenario 2 with 14% and 30% reduction for CD81 E219A and CD81 E219Q, respectively (Fig. 6F). Thus, E219 in the fourth CD81 transmembrane region is contributing to the replication-enhancing effect of CD81 and to the seemingly critical colocalization of CD81 with plasma membrane replication compartments. Notably, CD81 E219 is conserved across alphavirus-susceptible species (Fig. S3). Taken together, our results suggest that CD81 enhances CHIKV infection through a mechanism requiring cholesterol binding.

10.1128/mbio.00731-22.3 FIG S3 Alignment of CD81 orthologues from alphavirus-susceptible species. All protein domains are highlighted according to human CD81 protein domain assignments. Cyto, cytoplasmic; TMD1-4, transmembrane domains 1 to 4; SEL, small extracellular loop; LEL, large extracellular loop. Amino acids highlighted in red show differences between human and macaque or human and mouse CD81. Highlighted in blue is the cholesterol-binding glutamate residue located in transmembrane domain 4. Download FIG S3, TIF file, 2.0 MB. Copyright © 2022 Lasswitz et al. 2022 Lasswitz et al. https://creativecommons.org/licenses/by/4.0/ This content is distributed under the terms of the Creative Commons Attribution 4.0 International license.

### CD9 can partially substitute CD81 as chikungunya virus host factor.

**FIG 7:**
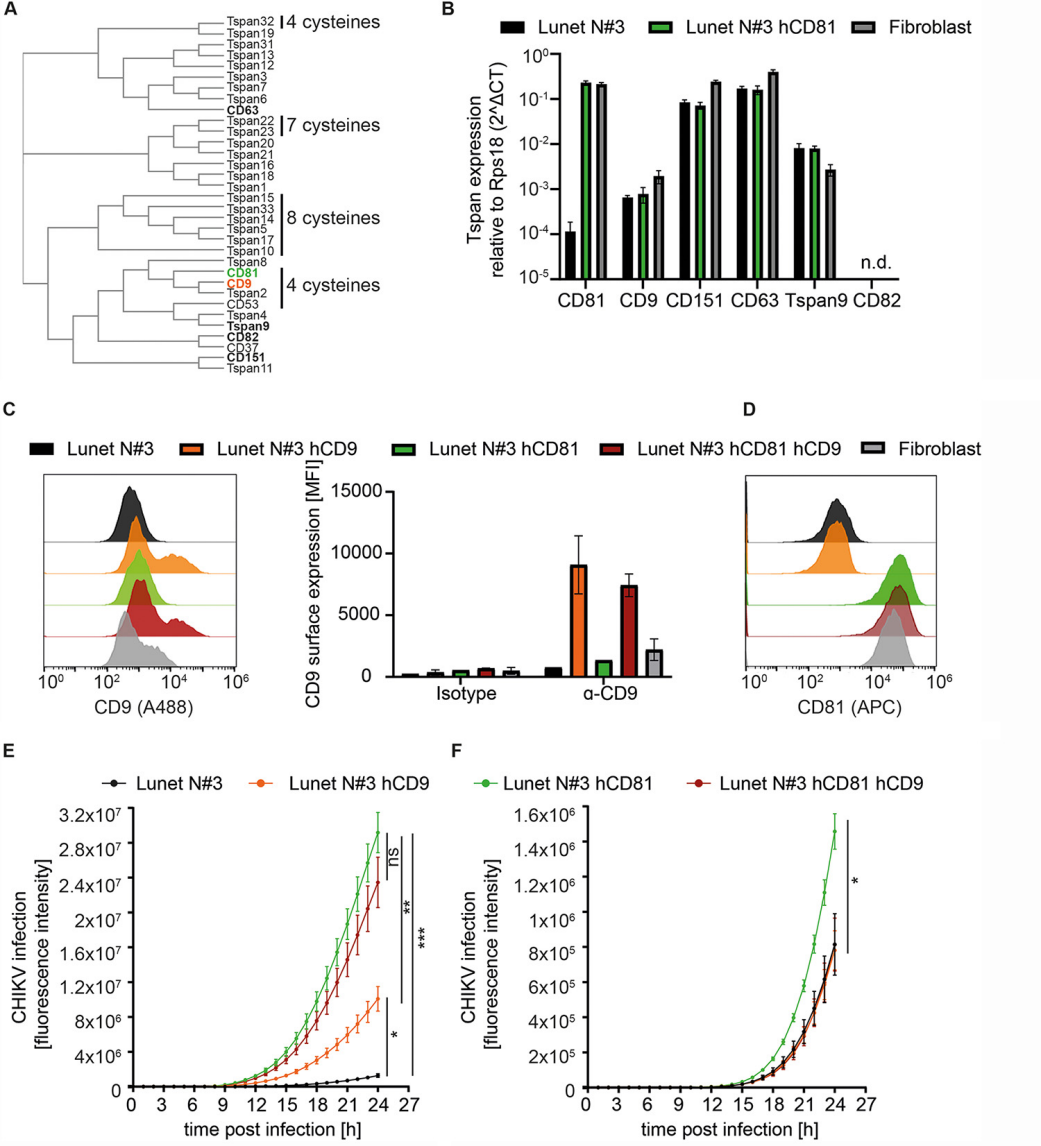
CD9 can partially substitute CD81. (A) Homology tree of the 33 human tetraspanins generated by protein sequence alignment using Clustal Omega. Tetraspanins highlighted in bold are described as virus host factors. CD81 is highlighted in green and CD9 in orange. The numbers of cysteine residues in the large extracellular loop different from 6 are highlighted. (B) The mRNA expression levels of tetraspanins known to be involved in pathogen infection were determined in hepatoma cells lacking or expressing CD81 as well as in dermal fibroblasts by qRT-PCR. n.d., not detectable. (C) CD9 surface staining of the indicated cell lines shown as representative histograms (left panel) or mean fluorescence intensity (MFI) (right panel). (D) CD81 surface staining of the indicated cell lines. (E and F) Lunet N#3 cells expressing the indicated tetraspanin were infected with a GFP reporter CHIKV (ECSA genotype; MOI, 5) (E) or mCherry reporter CHIKV (Asian genotype; MOI, 0.1) (F). Infection was followed over time using the IncuCyte S3 imaging platform and quantified as total GFP or mCherry object integrated intensity (GCU × μm^2^/image). Data are represented as the mean ± SD of three (B) or two (C) biological replicates, each performed in duplicates. (E and F) Data shown as the mean ± SEM of five (E) or three (F) biological replicates, each performed in triplicates. Statistics are based on two-way ANOVA with Turkey’s multiple-comparison test. *, *P* < 0.05; **, *P* < 0.01; ***, *P* < 0.001.

Tetraspanins are evolutionarily conserved proteins known to play a role in pathogen infections (26). For some viruses, tetraspanins function in a nonredundant manner e.g., CD81 for HCV entry, whereas for other viruses, tetraspanins may act redundantly e.g., CD81, CD9, and CD151 in HCMV entry (56). Since we observed a strong dependency of CD81 in hepatoma cells and a milder effect in fibroblasts, we asked if the expression pattern of tetraspanin family members differs between the two cell types and if another tetraspanin may serve as a host factor in fibroblasts. To date, 33 tetraspanins are described in humans (Fig. 7A). From those described to play a role in virus infection, we determined the mRNA expression level in hepatoma cells lacking or expressing CD81 as well as in dermal fibroblasts (Fig. 7B). Lunet N#3 hCD81 cells and fibroblasts express comparably high levels of CD81, while Lunet N#3 cells, as expected, express low CD81 levels. In contrast, CD9 showed low expression levels in all three cell lines and was 2.5-fold higher in fibroblasts than in the hepatoma cells. The mRNA expression levels of CD151 and CD63 were comparable between Lunet cells lacking or expressing CD81 and again slightly higher in fibroblasts. In contrast, Tspan9 expression levels were slightly lower in fibroblasts, and CD82 was not detectable in any of the three cell lines. As CD9 is the closest homolog of CD81 and showed only low expression levels in hepatoma cells, but slightly higher expression levels in fibroblasts, we decided to investigate whether CD9 can also function as CHIKV host factor. To this end, we overexpressed CD9 in Lunet cells lacking or expressing CD81 and confirmed the expression by CD9 surface staining and flow cytometry analysis (Fig. 7C). In addition, we confirmed that the CD81 expression remained constant upon CD9 expression (Fig. 7D). Lastly, we infected the single or double tetraspanin-expressing cell lines with a GFP-reporter CHIKV (ECSA genotype; MOI, 5) and followed infection over time using the IncuCyte S3 imaging platform. As expected, CD81 expression resulted in a significant increase of CHIKV infection, and CD81-CD9 coexpression revealed a similarly strong enhancement of CHIKV permissiveness (Fig. 7E). In addition, CD9 expression in the absence of CD81 led to a significant enhancement of CHIKV infection compared to Lunet N#3 cells. However, CHIKV infectivity in Lunet cells expressing CD81 was still significantly higher than infectivity in cells expressing CD9. Interestingly, an Asian genotype of CHIKV, which we showed to be less dependent on CD81 (Fig. 1C), only infected CD81-expressing cells better than parental cells, but not CD9 expressing cells (Fig. 7F). Moreover, coexpression of CD81 and CD9 led to similarly low infection rates with this Asian genotpye as observed in Lunet cells lacking CD81 and CD9 or Lunet cells expressing CD9 only. Together, our data show that CD9 can partially substitute CD81 as a host factor but has no additive or synergistic effect on CHIKV permissiveness. In addition, CD9 only enhanced infection of the CHIKV ECSA genotype, which is highly dependent on CD81.

## DISCUSSION

Tetraspanins are transmembrane proteins, which form homo- and heterodimers as well as complexes with other proteins and lipids, thereby shaping distinct membrane microdomains. Here, we identify the tetraspanin CD81 as a host factor for CHIKV and four other alphaviruses from three distinct genetic complexes. CHIKV is transmitted via mosquitoes and replicates during the preacute phase in dermal fibroblasts, followed by dissemination to the liver, lymph nodes, spleen, muscle, and joint tissues (1, 57). The liver is, together with lymph nodes and spleen, a primary site of infection during the acute phase with peak viremia (58–61). Here, we show that CD81 expression in human hepatocytes renders cells highly permissive to CHIKV infection and confirm the finding in dermal fibroblasts using CRISPR/Cas9 genetic ablation. In sum, using three orthogonal methods, namely, gain of function, RNA interference, and CRISPR/Cas9 loss of function experiments, we demonstrate in two relevant cell types that CD81 is a host factor for CHIKV.

Notably, the dependency on CD81 differs between CHIKV strains, with the globally circulating LR2006-OPY1 (Indian Ocean sublineage of the ECSA genotype) being most dependent on CD81. Such strain-dependent host factor usage was also shown for Mxra8, with strains of Asian and West African genotypes being highly dependent and ECSA strains being less dependent on Mxra8 (11). For the replication factor FHL-1, strains of the Asian, ECSA, or Indian Ocean lineage show strong dependency, as opposed to less dependent strains of the West African genotype (14). While the open reading frames of different CHIKV strains are highly conserved, the 3′ and 5′ untranslated regions (UTRs) are quite variable, resulting in differential fitness and ability to adapt to new environments (62–64). As secondary structure elements in the UTRs are critical for viral genome replication, the differential dependency on CD81 observed in our study could reflect strain-specific differences in the UTRs (65). In addition, the nucleotide substitution rate is higher in epidemic lineages (Asian and Indian Ocean lineages) than in enzootic lineages (West African and ECSA) (64), which could lead to differential selection pressure and evolution of host factor usage. Moreover, to our knowledge, it is not described yet whether all genotypes equally depend on spherule formation for genome replication.

Alphaviruses infect a wide range of vertebrate and mosquito species, with each virus having a distinct spectrum of reservoir and transmitting host species. CHIKV is thought to reside in primates and can infect mice experimentally (1, 60, 66). Here, we show that murine CD81 can substitute human CD81 and function as a CHIKV host factor. This is in stark contrast to the host factor function of CD81 during HCV infection, in the context of which, murine CD81 fails to substitute human CD81 (42, 67, 68). Overall, mouse CD81 shares 92% amino acid identity with human CD81. While the CD81 large extracellular loop is the species tropism-defining domain for HCV, our results indicate that the cholesterol-binding regions in the CD81 transmembrane domains are critical for CHIKV infection. In line with this notion, the cholesterol-binding residues are conserved between human and murine CD81 (55). To this end, it would be interesting to investigate whether CD81 orthologues from mosquitoes, which also harbor a cholesterol-binding glutamate residue in the fourth transmembrane domain, can function as CHIKV host factors. Notably, the Aedes albopictus tetraspanin C189 plays a role in cell-to-cell spread of dengue 2 virus (69, 70). In sum, our current study suggests that CD81 may be a cross-species host factor for alphaviruses in vertebrates.

A remarkable feature of CD81 is its involvement at different life cycle steps of various viruses and bacteria (26, 71, 72). For instance, CD81 functions as an HCV receptor via interaction of its large extracellular loop with the E2 glycoprotein of the virus (33, 34). Moreover, CD81 enhances reverse transcription of HIV-1 by direct interaction with SAM domain and HD domain-containing protein1 (SAMHD1), which increases the deoxynucleotide triphosphates (dNTPs) needed for reverse transcription (36). On the other hand, some studies show that HIV-1 buds from TEMs comprising CD81, CD9, and CD63 and that the HIV-1 envelope glycoprotein colocalizes with tetraspanins (41, 73, 74). Three independent studies suggest that CD81 expression levels inversely correlate with infectivity of released HIV-1 particles (75–77), and recent work demonstrates that tetraspanins are important for protein partitioning at the HIV-1 budding site (41). In addition, cholesterol and sphingolipids participate in protein partitioning at HIV-1 budding sites (78). Such close interplay of proteins and lipids in shaping bend plasma membrane structures could explain our observation that CD81 and its cholesterol-binding site are critical for CHIKV replication.

Most alphaviruses, including CHIKV, replicate their RNA genomes in virus-induced plasma membrane compartments termed spherules (37, 38). These spherules are plasma membrane buds, which presumably shield the viral RNA from innate immune recognition. Given the reported role of CD81 in shaping budding sites of HIV-1 (41), it is plausible that CD81 participates in the formation of spherules at the plasma membrane. This is in line with our observation that cells expressing CD81 show a distinct localization of viral dsRNA intermediates near the plasma membrane (overlapping with ZO-1), while cells lacking CD81 display a more diffuse intracellular staining. In addition, CD81 and dsRNA colocalize at the plasma membrane. To confirm that CD81 is part of the spherule membrane, immunogold labeling and electron microscopy of CHIKV-infected cells will be required. In this study, we show that CD81 is required for efficient replication of CHIKV genomes and for localization of dsRNA intermediates at the plasma membrane.

Concerning the mode of action of CD81-enhanced CHIKV replication, our data hint toward a CD81-dependent regulation of membrane cholesterol content in the spherule microdomains. CD81 is a plasma membrane protein harboring a cholesterol-binding pocket with E219 as an important residue. Mutations in E219, i.e., E219A and E219Q, show a 2-fold-reduced cholesterol-binding capacity (54), and we observed a concomitant reduction in CHIKV infection levels in cells expressing these variants instead of wild-type CD81. This demonstrates that the cholesterol-binding site of CD81 plays a critical role in CHIKV infection. In line with this, the signal for dsRNA replication intermediates was less prominent at the plasma membrane in cells expressing the cholesterol-binding mutants of CD81. In addition, we observed a reduction in CD81-dsRNA colocalization for the cholesterol-binding mutants compared to wild-type CD81. Notably, CD81 promotes invasion of the malaria sporozoites from Plasmodium yoelii and Plasmodium falciparum via a cholesterol-dependent mechanism (48, 79). Here, we observe that CD81 is dispensable for CHIKV entry and is instead required during later steps of the infection cycle, i.e., RNA replication. Intriguingly, alphavirus RNA replication uniquely takes place at plasma membrane buds (spherules). As the bending of the plasma membrane requires the presence of cholesterol and possibly sphingolipids, we hypothesize that one function of CD81 is to provide the correct lipid environment in the spherules.

**FIG 8:**
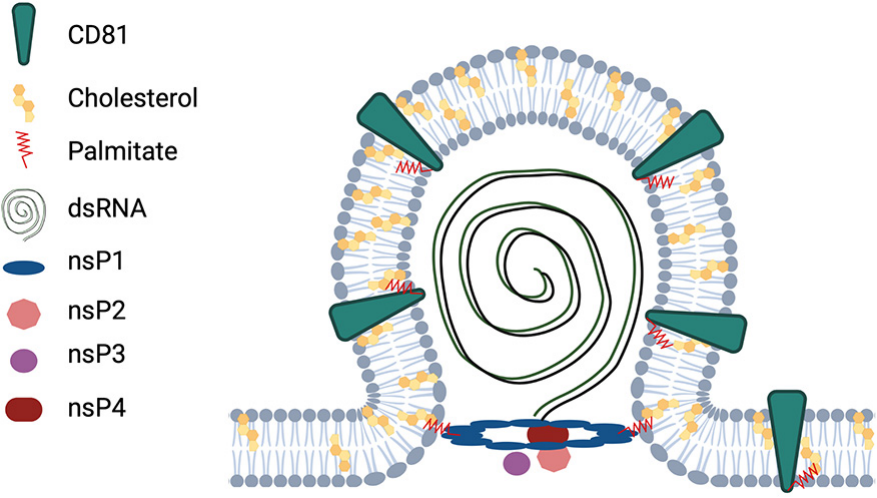
Proposed model for the role of CD81 in CHIKV infection. CD81 is a plasma membrane protein that binds to cholesterol and has a cone-like topology. CHIKV nsPs form replication compartments at the plasma membrane, termed spherules, where dsRNA is synthesized during active replication. Our data suggest that CD81 promotes membrane curvature, thereby facilitating CHIKV replication at the plasma membrane. Illustration created with BioRender.com and modified from reference 80 and 105.

A second important biophysical feature of CD81 is its shape, with the four transmembrane domains forming a cone-like topology opening up to the extracellular leaflet of the membrane (54). Such conically shaped proteins are thought to promote membrane bending (80). Thus, we propose that CD81, in addition to enriching cholesterol at the CHIKV replication complex, also provides an appropriate protein topology for spherule formation (Fig. 8). Whether other proteins with a similar topology could consequently substitute CD81 remains to be analyzed. The tetraspanin CD9 has a cone-like topology similar to that of CD81 and can induce membrane bending (81). Here, we could gain initial insight if CD9, the closest homolog of CD81 could substitute CD81. Interestingly, CD9 can partially increase CHIKV infection in cells lacking CD81. In line with this, it is known that tetraspanins can have redundant function; for instance, CD81, CD63, and CD151 can substitute for each other during HCMV entry (56). However, we only observed a partial substitution of the CD81 function by CD9 for the CHIKV ECSA genotype but not for the Asian genotype, which we show to be less dependent on CD81 overall. This suggests that different CHIKV genotypes differentially interact with tetraspanin-enriched microdomains. A full survey of the role of all 33 tetraspanin members in CHIKV infection with different genotypes is part of ongoing investigations.

A possible specificity of CD81 could be mediated by interaction with CHIKV nonstructural proteins located at the replication complex. A prime candidate for such an interaction is the viral methyl/guanylyltransferase nsP1, which anchors CHIKV replication complexes to cholesterol-rich microdomains through palmitoylated cysteine residues (39). Palmitoylation-deficient nsP1 fails to target to detergent-resistant membrane microdomains (DMRs) and leads to a strong reduction in CHIKV replication (39, 82, 83). The same study demonstrated a colocalization of CHIKV nsP1 and CD81, supporting a possible link between both proteins. While complex formation of these proteins remains to be demonstrated, it is important to note that SINV nsP1 only moderately segregates to DRMs (39) at the plasma membrane (37, 84). In line with this, palmitoylation-deficient SINV nsP1 does not reduce viral titers but, instead, delays infection kinetics (85, 86). In our study, we also observe a significant dependency of SINV on CD81 for efficient replication. This indicates that CD81 may promote replication via several distinct pathways, only some of which may be shared between alphaviruses from different complexes.

Finally, cholesterol binding is thought to change the CD81 conformation (54, 87), thereby affecting interactions with other human proteins. Molecular modeling suggests that binding of cholesterol results in a closed CD81 ectodomain conformation, while unbound CD81 shows a more open ectodomain conformation (87). This conformational switch is thought to influence HCV entry, which depends on the ability of CD81 to bind cholesterol (55, 87). Importantly, the majority of CD81 protein interactions are mediated by the ectodomain. Hence, the switching between the open and closed conformation likely affects CD81 protein interactions. Whether CD81 interactions with additional host proteins affect CHIKV replication needs to be further investigated.

Using CRISPR/Cas9 technology, we here show that CD81 is required not only for CHIKV infection in human hepatocytes, but also in dermal fibroblasts, suggesting that CD81 may be a broad host factor in different viral target tissues. Importantly, CD81 is dispensable for infection with coronaviruses and arenaviruses tested in this study, demonstrating that CD81 is an alphavirus-specific replication factor. However, coronaviruses use tetraspanin CD9 as an entry cofactor (29, 30), and the arenavirus Lujo virus hijacks CD63 for glycoprotein-mediated membrane fusion (88). Thus, viruses may have evolved to use distinct tetraspanins and, consequently, distinct membrane microdomains to orchestrate different life cycle steps. In this study, we identify the tetraspanin CD81 as a critical alphavirus host factor that orchestrates virus replication at the plasma membrane.

## MATERIALS AND METHODS

### Cell culture.

The human hepatoma cell lines Huh-7.5 (89), HepG2, HepG2 hCD81, HepG2 mCD81, Lunet N#3, Lunet N#3 hCD81, Lunet N#3 mCD81 (42), Lunet N#3 hCD81 E219A, and Lunet N#3 hCD81 E219Q (55); human dermal fibroblasts (kindly provided by F. Pessler, Twincore, Hannover, Germany), and 293T and BHK-21 cells were cultured at 37°C and 5% CO2 in Dulbecco’s modified Eagle’s medium. The medium was supplemented with 10% fetal calf serum (FCS), 2 mM l-glutamine, 0.1 mM nonessential amino acids, and 1% penicillin/streptomycin. Lunet N#3 hCD81, Lunet N#3 mCD81, and derived cell lines, as well as HepG2 hCD81 and HepG2 mCD81 were additionally cultured in the presence of 5 μg/mL blasticidine. Primary mouse fibroblasts were cultured in RPMI 1640 medium containing 10% FCS, 50 μM 2-mercaptoethanol, 100 μM asparagine, 2 mM glutamine, and 1% penicillin/streptomycin. All cell lines were tested regularly for *Mycoplasma* contamination.

### Generation of hepatoma cells expressing CD9.

Lunet N#3 cells and Lunet N#3 hCD81 cells stably expressing CD9 were generated by lentiviral gene transfer as described before (68). Briefly, 293T cells were cotransfected with pCMVΔR8-74, pczVSV-G, and pTRIP-CD9 (5 μg of each). Supernatants containing lentiviral particles were harvested 48 h and 72 h posttransfection and filtered through a 0.45-μm-pore-size filter. To stabilize the particles and increase transduction efficiency, Polybrene and HEPES were added to a final concentration of 5 μg/mL and 20 mM, respectively. Lunet N#3 cells and Lunet N#3 hCD81 cells were transduced with the lentiviral particles for 72 h at 37°C. Expression of CD9 was confirmed by antibody surface staining and flow cytometry analysis as described below.

### Viruses and pseudoparticles.

The infectious clones of CHIKV LR2006-OPY1 (ECSA genotype) and CHIKV 37997 strain (West African genotype) expressing GFP under the control of a subgenomic promoter and CHIKV 181/25 strain (Asian genotype) encoding Nano-luciferase (Nluc) or mCherry (fused to the N terminus of E2 protein) have been described previously (90–94). The alphaviruses VEEV TC-83 strain expressing EGFP under a subgenomic promoter, SINV Toto 1011 with in-frame firefly luciferase insertion in nsP3, and wild-type RRV strain T48 have previously been reported (95–97). Infectious virus was produced by *in vitro* transcription followed by electroporation of RNA into BHK-21 cells. Supernatant was collected 48 h after electroporation and titrated on 293T or BHK-21 cells. SFV EGFP-nsp3 virus from virulent strain SFV4 was produced by electroporation of the plasmid pSFV(3H)4-EGFP into BHK-21 as previously described (98).

CHIKV single-round infection particles (53) were generated by cotransfection of CHIKV genomic RNA containing an EGFP reporter and two helper RNAs expressing the CHIKV capsid protein or the residual structural proteins (E3, E2, 6K, E1) into BHK-21 cells by electroporation. The plasmids pChikRepl, pChikHelper-E, and pChikHelper-C to generate the single-round infection particles were kindly provided by Andres Merits. Single-round infectious particles were harvested 2 days postelectroporation. For CHIKV replicon assays, only the genomic RNA containing the nonstructural proteins and an EGFP reporter were transfected into target cells using Lipofectamine 2000 reagent.

HCV-G2a encoding a Renilla luciferase (JcR2a) was *in vitro* transcribed and electroporated into Huh-7.5 cells. After 48-h, 72-h, and 96-h supernatants were harvested and titrated on Huh-7.5 cells. The recombinant human coronavirus strain HCoV-229E encoding a *Renilla* luciferase (kindly provided by V. Thiel, University of Bern, Switzerland) (99) was amplified in and titrated on Huh-7.5 cells.

The vaccine JUNV strain Candid#1 (kindly provided by Stefan Kunz, CHUV Lausanne, Switzerland) was passaged in and titrated on Vero cells.

To study viral entry, lentiviral pseudoparticles harboring glycoproteins of CHIKV, VSV, or HCV or without any viral envelope glycoprotein and encoding a firefly luciferase were generated as described elsewhere (100). Target cells were transduced with the pseudoparticles for 72 h, the cells were lysed in water, and luciferase signal was quantified using coelenterazine substrate and a plate luminometer (Berthold).

### Infection assays.

For infection with CHIKV expressing a GFP or mCherry reporter, cells were infected for 8 h, 24 h, or 48 h with the indicated MOIs (based on titration on 293T cells), trypsinized, and fixed with 2% paraformaldehyde (PFA) for 90 min at room temperature (RT). GFP- or mCherry-positive cells were analyzed using an Accuri C6 flow cytometer (BD) or a Sony SA3800 spectral cell analyzer (Sony). For infection with Nluc CHIKV, cells were infected for 2 h to 10 h followed by luciferase measurement in cell lysates using coelenterazine substrate and a plate luminometer (Berthold). In order to determine CHIKV permissiveness of hepatoma cells expressing CD81 or CD9, cells were infected with GFP (MOI, 5) or mCherry (MOI, 0.1) reporter CHIKV. Infection was followed over time with the IncuCyte S3 imaging platform (Sartorius) using ×10 magnification and acquisition of three images per 96-well plate. Data were analyzed using the manufacturer’s basic analyzer tool.

To assess the dependency of other alphaviruses to CD81 expression, Lunet #N3 or Lunet #N3 hCD81 cells were infected with reporter-expressing VEEV or SINV at the indicated MOIs (based on BHK-21) for 24 h and infection measured by flow cytometry or bioluminescence, respectively. For SFV and RRV, Lunet #N3 or Lunet #N3 hCD81 cells were infected for 2 h at the specified MOIs before removal of virus inoculum and addition of fresh medium. Then, 24 h postinfection, supernatants from infected cells were collected and titrated on BHK-21 by standard plaque assay.

For JUNV infection, cells were infected 48 h after seeding with an MOI of 1 (based on Vero cells). The viral inoculum was removed after 90 min, and fresh DMEM medium was added. Cells were trypsinized and fixed with 2% PFA at 24 h, 48 h, and 72 h after infection. For flow cytometric analysis, cells were permeabilized in FACS buffer (1% FCS in PBS) containing 0.1% saponin (permeabilization buffer) for 20 min on ice, followed by staining with an anti-NP antibody (BEI Resources; clone MA03-BE06, 1:1,500) for 1 h on ice. After one washing step with FACS buffer, cells were incubated with an Alexa 647-conjugated secondary antibody for 30 min on ice. After two additional washing steps, cells were analyzed using the Sony SA3800 spectral cell analyzer (Sony).

For CoV-229E infection, 24 h after seeding, cells were infected with an MOI of 0.1 and 0.01. Infection was measured by *Renilla* luciferase activity in cell lysates 48 h after infection.

### CD81 antibody blocking experiment.

For CD81 antibody blocking experiments, adherent Lunet N#3 hCD81 cells were incubated with 5 μg/mL anti-CD81antibody (clone Js-81, BD) or isotype control for 30 min at 37°C. Afterward, cells were infected with a GFP-encoding CHIKV (ECSA genotype) or HCV-JcR2a in the presence of CD81 antibody or isotype control for 4 h at 37°C. Cells were washed twice with phosphate-buffered saline (PBS), fresh medium was added, and infection was stopped after 24 h for CHIKV and 48 h for HCV. For CHIKV, GFP-positive cells were measured by flow cytometry (Accuri C6, BD) as readout for infection. HCV infectivity was determined by luciferase activity using coelenterazine as the substrate and a plate luminometer (Berthold).

### Flow cytometry and immunofluorescent microcopy.

For analysis of dsRNA, cells were infected with E2-mCherry reporter CHIKV (Asian genotype) for 9 h at an MOI of 50 (based on titration on 293T cells). For flow cytometry analysis, cells were trypsinized and fixed with 2% PFA for 90 min at RT. Cells were permeabilized in permeabilization buffer (0.1% saponin, 1% FCS in PBS) for 20 min on ice followed by incubation with 10 μg/mL monoclonal anti-dsRNA antibody (clone J2, SCICONS) for 60 min on ice (in permeabilization buffer). After a brief wash, 2 μg/mL Alexa 488-conjugated secondary antibody was added for 30 min on ice (in permeabilization buffer). Cells were washed twice before analysis with a Sony SA3800 spectral cell analyzer (Sony). Data analysis was performed using FlowJo.

For immunofluorescence analysis, cells were washed three times after PFA fixation, blocked for 10 min with 0.5% bovine serum albumin (BSA) in PBS, and surface stained for ZO-1 (ZO1-1A12, Thermo Fisher; 5 μg/mL) or CD81 (EPR21916, abcam; 6 μg/mL) overnight at 4°C in PBS with 0.5% BSA. The next day, cells were stained for 1 h at RT with 2 μg/mL secondary antibody (Alexa 647-conjugated) followed by permeabilization with 0.1% Triton X-100 in PBS for 4 min. Afterward, cells were stained with 5 μg/mL monoclonal anti-dsRNA antibody (SCICONS, clone J2) overnight at 4°C in PBS with 0.5% BSA. Secondary staining with 2 μg/mL Alexa 488-conjugated secondary antibody was performed for 1 h at RT. Cell nuclei were counterstained with DAPI (4′,6-diamidino-2-phenylindole; 300 nM, Life Technologies), and coverslips were mounted in Prolong Gold. Fluorescence microscopy images were acquired using an inverse confocal laser-scanning microscope (Olympus Fluoview 1000 or Leica TCS SP5) with ×60 or ×100 magnification lenses. Channels were read in sequential acquisition mode with a Kalman filter. To quantify localization of dsRNA, confocal images of cells stained with ZO-1 or CD81 and costained with dsRNA were acquired as described above. At least 50 infected cells per cell line were analyzed for ZO-1 and dsRNA costaining, and at least 68 were analyzed for CD81 and dsRNA costaining. For each infected cell, the dsRNA localization was analyzed blinded and independently by two investigators using the three different scenarios defined in Fig. 5D and 6D. The data are represented as averages (calculated from the counts determined by the two investigators).

To quantify CD81 surface expression on cells after siRNA knockdown or CRISPR/Cas 9 knockout, nonpermeabilized cells were stained with anti-CD81 antibody. In detail, cells were trypsinized, quenched, and stained with anti-hCD81-APC antibody (BD; JS-81, 10 μL per 5 × 10^5^ cells) or isotype control (BD; 10 to 20 μL per 5 × 10^5^ cells) for 30 min on ice in FACS buffer. Lunet N#3 mCD81and primary mouse fibroblasts were stained with 10 μg/mL anti-mCD81-PE antibody (Santa Cruz, EAT-2) for 30 min on ice in FACS buffer. To quantify CD9 surface expression, nonpermeabilized cells were stained with anti-CD9 antibody (clone SN4/C3-3A2, Ancell; 5 μg/mL per 5 × 10^5^ cells) for 1 h at 4°C, followed by secondary antibody staining (10 μg/mL Alexa 488-conjugated) for 30 min at 4°C. After staining, cells were washed twice with FACS buffer and analyzed by flow cytometry using an Accuri C6 (BD), Sony SA3800 spectral cell analyzer (Sony), or an Attune NxT cytometer (Invitrogen).

### Viral RNA transfection and replicon particle assay.

To analyze replication independent of entry, cells were transfected with either full-length Nluc-CHIKV RNA (Asian genotype) or a CHIKV replicon (ECSA genotype) containing the nonstructural proteins and an EGFP reporter. For transfection with full-length CHIKV RNA, cells were transfected with 0.4 μg RNA/well of a 96-well plate using Lipofectamine 2000 reagent according to the manufacturer’s instructions. After 2 h, 4 h, 8 h, and 10 h, cells were lysed, and luciferase activity was measured. For transfection with the CHIKV replicon, cells were transfected with 0.5 μg RNA/well of a 24-well plate. In parallel, cells were infected with single-round infection particles (see “Viruses and Pseudoparticles,” above). After 4 h, 8 h, 24 h, and 48 h, cells were trypsinized and fixed with 2% PFA for 90 min at RT. GFP-positive cells were analyzed using a Sony SA3800 spectral cell analyzer (Sony).

### CRIPSR/Cas9 knockout cell generation and validation.

CD81 knockout cells were generated using the CRISPR/Cas9 technique. The single guide RNA (sgRNA) targeting CD81 (TGGTGGTCTGCGGGTCATGG) and the scrambled, nontargeting sgRNA (CTAAGGTTAAGTCGCCCTCG) were used as described before (101). Briefly, sgRNAs were selected using CHOPCHOP (102) and cloned into pLenti CRISPR v2 ccdB as described in references 103 and 104. VSV-G lentiviral pseudoparticles were generated using standard procedures in 293T cells. Briefly, 293T cells were transfected with 5 μg pczVSV-G, 7.5 μg pcMVΔR8-74, and 11 μg pLenti CRISPR v2-derived plasmid using Lipofectamine 2000 reagent. After 24 h, medium was replaced with fresh DMEM containing 30% FCS. One day later, cell culture supernatant containing lentiviral particles was harvested. Human dermal fibroblasts were transduced with lentiviral particles for 24 h, followed by a second transduction for 24 h. After 48 h of the second transduction, cells were selected for Cas9 and sgRNA expression using hygromycin B. Surviving cells were characterized by antibody staining and flow cytometric analysis (see “Flow Cytometry and Immunofluorescent Microcopy,” above). Two independent batch populations were used for the experiments.

### Small interfering RNA (siRNA) knockdown experiments.

Huh-7.5 cells were transfected with a pool of three siRNAs targeting CD81 (siRNA ID s2722, s2723, s2724; Thermo Fisher Scientific) or a nontargeting siRNA as negative control (Silencer select negative control no. 2, Thermo Fisher Scientific) using Lipofectamine RNAiMAX reagent according to the manufacturer’s recommendations (Thermo Fisher Scientific). In short, Huh-7.5 cells were transfected with 12.5 nM final concentration of siRNAs in a 96-well plate. 24 h posttransfection a medium change was performed, followed by infection with a GFP CHIKV reporter (ECSA genotype) 48 h posttransfection. Then, 24 h postinfection, cells were trypsinized and fixed with 2% PFA for 90 min at RT. GFP-positive cells were analyzed by flow cytometry as described above. In parallel, Huh-7.5 cells were transfected with siRNAs and surface stained for CD81 48 h posttransfection to evaluate knockdown efficiency (see “Flow Cytometry and Immunofluorescent Microcopy,” above).

### qRT-PCR.

RNA was isolated from cell lysates of hepatoma cells and fibroblasts using the NucleoSpin RNA kit (Macherey-Nagel) according to the manufacturer’s instructions. The total RNA concentration was determined using a NanoDrop spectrophotometer. A total of 500 ng of isolated RNA was reverse transcribed using PrimeScript RT master mix (TaKaRa) according to the manufacturer’s protocol. In order to analyze the tetraspanin transcript expression in hepatoma cells and fibroblasts, the cDNA was then used for quantitative PCR (qPCR) using SYBR premix *Ex Taq* II (TaKaRa). Per reaction, 25 ng of cDNA was mixed with 1× SYBR premix *Ex Taq* II and each primer pair (0.6 μM final concentration). The reverse transcription-quantitative PCR (qRT-PCR) was conducted using the LightCycler R480 (Roche). The mRNA expression levels were calculated by relative quantification using the Δ*CT* method with Rps18 as the reference gene. Primer pairs were selected from the Harvard Primer Bank for CD81 (forward: TTCCACGAGACGCTTGACTG; reverse: CCCGAGGGACACAAATTGTTC), for CD9 (forward: CCTGCTGTTCGGATTTAACTTCA; reverse: TGGTCTGAGAGTCGAATCGGA), for Tspan9 (forward: GTGGTGTCACTGACTACACAG; reverse: TTCTCCACAAAGGCGTGGTG), for CD151 (forward: ATGGGTGAGTTCAACGAGAAGA; reverse: GCAGGCTGATGTAGTCACTCT), for CD82 (forward: TGTCCTGCAAACCTCCTCCA; reverse: CCATGAGCATAGTGACTGCCC), CD63 (forward: ATGCAGGCAGATTTTAAGTGCT; reverse: GTTCTTCGACATGGAAGGGATTT), and for Rps18 (forward: ATCACCATTATGCAGAATCCACG; reverse: GACCTGGCTGTATTTTCCATCC).

### Mice.

Mice (C57/BL6 mice; Charles River Laboratories) were housed in the animal facility at Helmholtz Centre for Infection Research in Braunschweig, Germany. CD81^−/−^ mice (Cd81^tm1Lvy^; MGI:2180805) (44, 48) were bred as heterozygous to generate from the same breeding CD81^−/−^ and their respective littermate wild-type control mice. Heterozygous mice were kindly provided by Shoshana Levy, Olivier Silvie, and Eric Rubinstein. Mice were genotyped by PCR of ear DNA. All animal care and procedures were in accordance with institutional guidelines and European regulations.

### Isolation of primary mouse fibroblast from ear tissue.

The isolation of primary mouse fibroblast from ear tissue was performed as described in detail in reference 45. Briefly, CD81^−/−^ mice and wild-type littermates were euthanized according to institutional guidelines. Ears were cut, disinfected with 70% ethanol, and air-dried under a cell culture cabinet. After removal of hair, ears were cut into small pieces in RPMI 1640 medium containing 10% FCS, 50 μM 2-mercaptoethanol, 100 μM asparagine, 2 mM glutamine, and 1% penicillin/streptomycin solution. Cut tissue was digested using collagenase d-pronase solution for 90 min at 37°C and 200 rpm. Digested tissue was ground through a 70-μM cell strainer into a cell culture dish. The cell suspension was collected, centrifuged for 7 min at 580 × *g* at 4°C, and washed twice with medium. The cells were resuspended in medium, added to a 10-cm culture dish, and supplemented with 10 μL amphotericin B solution (stock solution, 250 μg/mL). The cells were incubated at 37°C and 5% CO_2_. After 3 days, cells had attached to the culture dish, and medium change was performed. Then, 4 to 5 days after isolation, cells were used for experiments.

### Immunolabeling and imaging of mouse tissue.

Native murine liver and muscle tissues from wild-type and CD81 knockout mice were embedded in Tissue-Tek O.C.T. compound (Sakura Europe) and snap-frozen in liquid nitrogen. Frozen sections (2 to 3 μm thick) were mounted on Superfrost Plus slides (Thermo Scientific) and dried at RT for 20 min prior to acetone fixation for 10 min. They were then dried again for 20 min at RT. After washing in PBS for 30 min, sections were incubated with a directly conjugated anti-mCD81-PE antibody (Santa Cruz, EAT-2) diluted 1:50 in PBS with 1% BSA (Sigma-Aldrich) at 4°C overnight in the dark. Sections were washed three times for 5 min in PBS with 0.1% Triton-X (Sigma-Aldrich) and two times with PBS only before washing for 1 min in distilled water. Costaining of nuclei was performed with bisbenzimid (H 33258, Sigma-Aldrich) diluted 1:100 in distilled water by incubation for 10 min at RT in the dark before washing for 1 min in distilled water. Coverslips were mounted with Dako fluorescent mounting medium (Agilent Technologies). Stained sections were analyzed, and images were obtained using a digital fluorescence microscope (Keyence BZ-9000) and BZ-II Analyzer software. The 4×, 10×, and 20× lens objectives were used for imaging. Preparation of image plates and digital zoom were performed with Adobe Photoshop CS6.

### Alignment and homology analysis.

Amino acid sequences for tetraspanins were retrieved from UniProt (human, ID P60033; macaque, ID F6WY45; mouse, ID P35762; mosquito, Q16J92) and aligned using the Clustal Omega algorithm (version 1.2.4). Protein domains were retrieved from UniProt and highlighted manually.

### Statistical analysis.

Experiments were conducted in at least three biological replicates with three technical replicates each unless stated otherwise. Graphical representation and statistical analysis were performed with GraphPad Prism versions 7 and 9. Unless stated differently, results are presented as the mean ± the standard deviation (SD) of three biological replicates. Statistical significance was determined using an unpaired two-tailed *t* test or two-way analysis of variance (ANOVA) with Sidak’s multiple-comparison test or Dunnett’s multiple-comparison test. Figures were generated with Adobe Illustrator version 25.0.1.
