# Supplementary figures and images for: The Tetraspanin CD81 Is a Host Factor for Chikungunya Virus Replication
Source: mBio. 2022 May 25;13(3):e00731-22. doi: 10.1128/mbio.00731-22 (PMC9239085; doi:10.1128/mbio.00731-22)

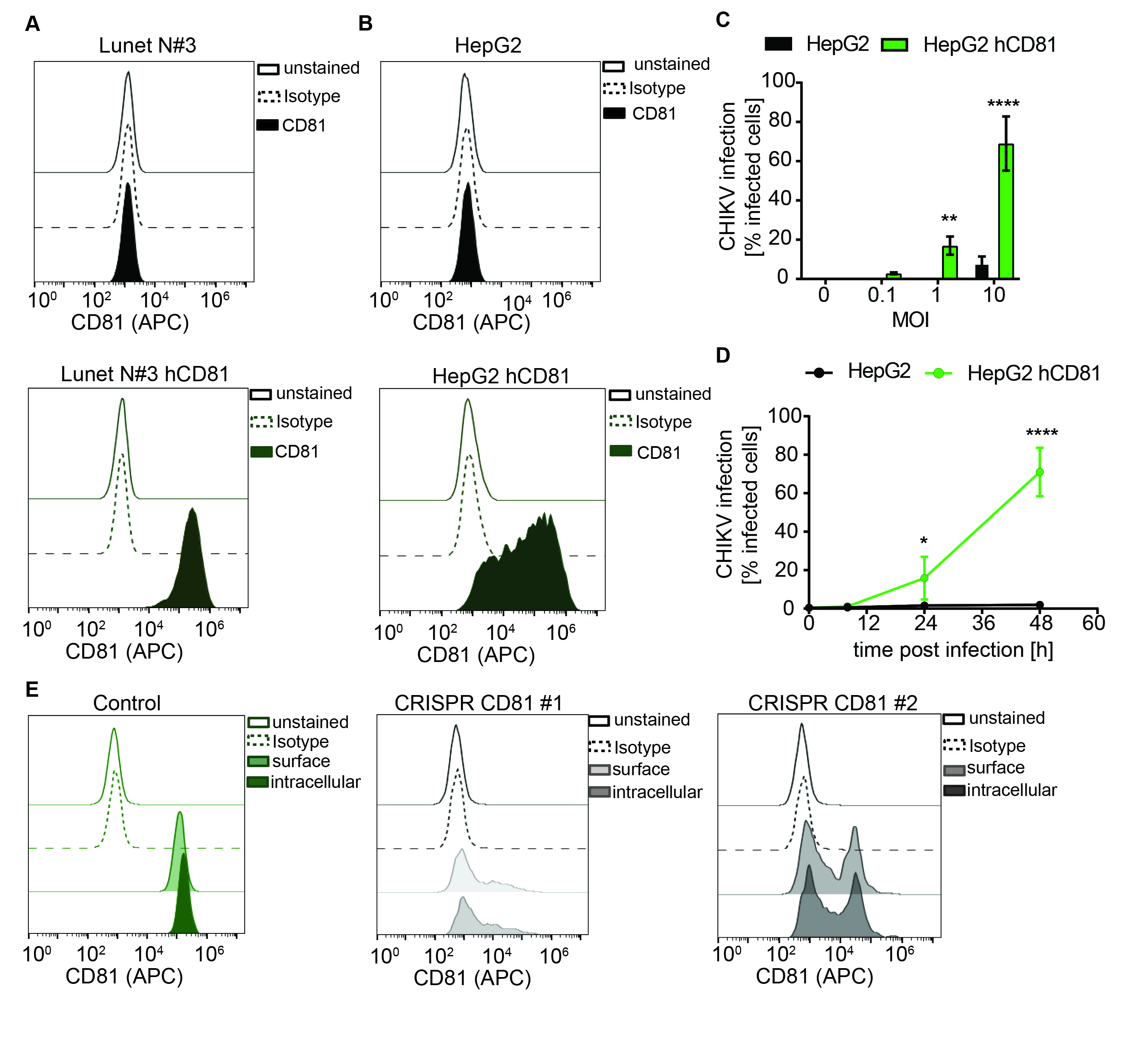

Supplement: FIG S1 [file mbio.00731-22-s0001.tif]

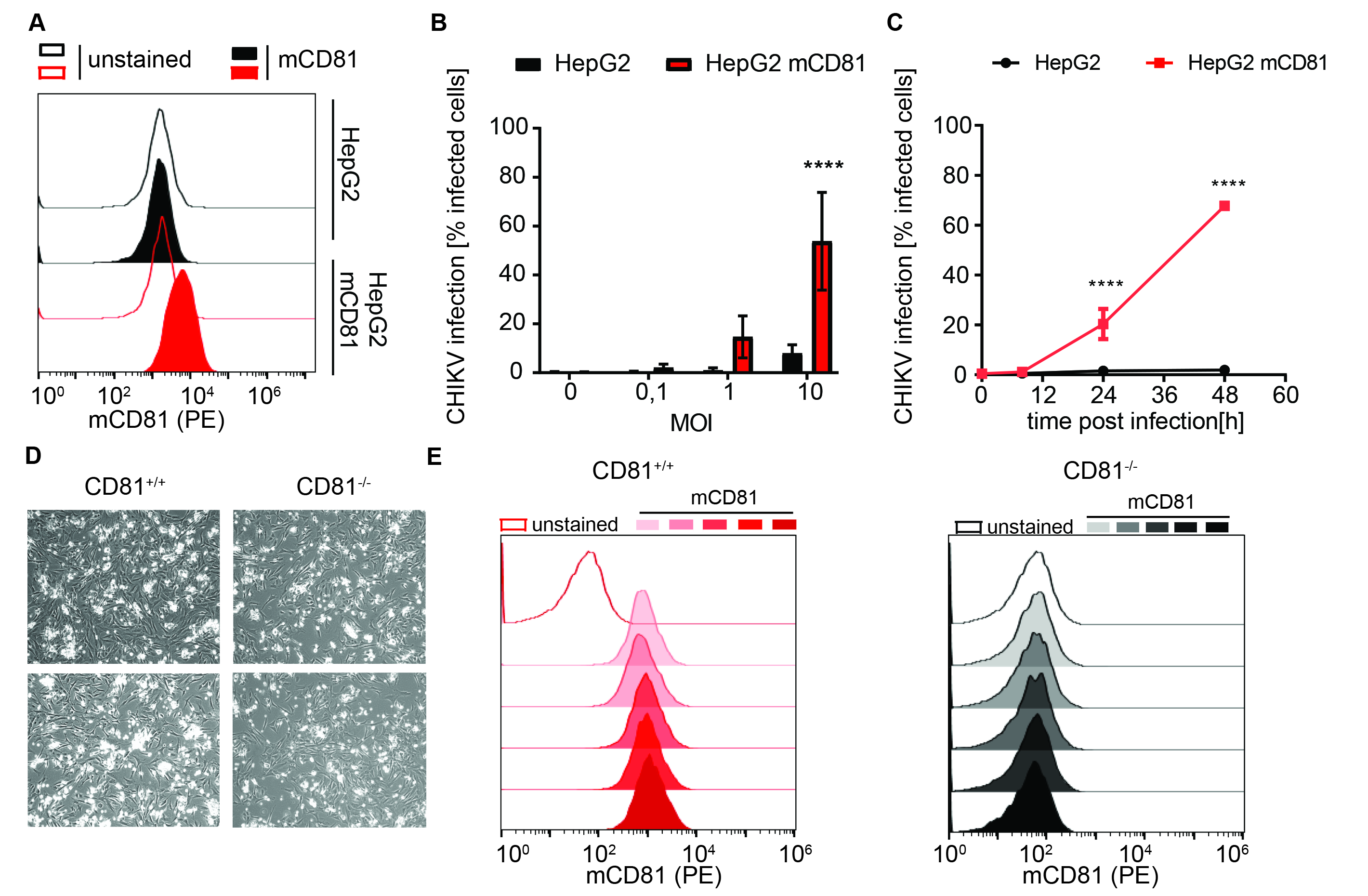

Supplement: FIG S2 [file mbio.00731-22-s0002.tif]

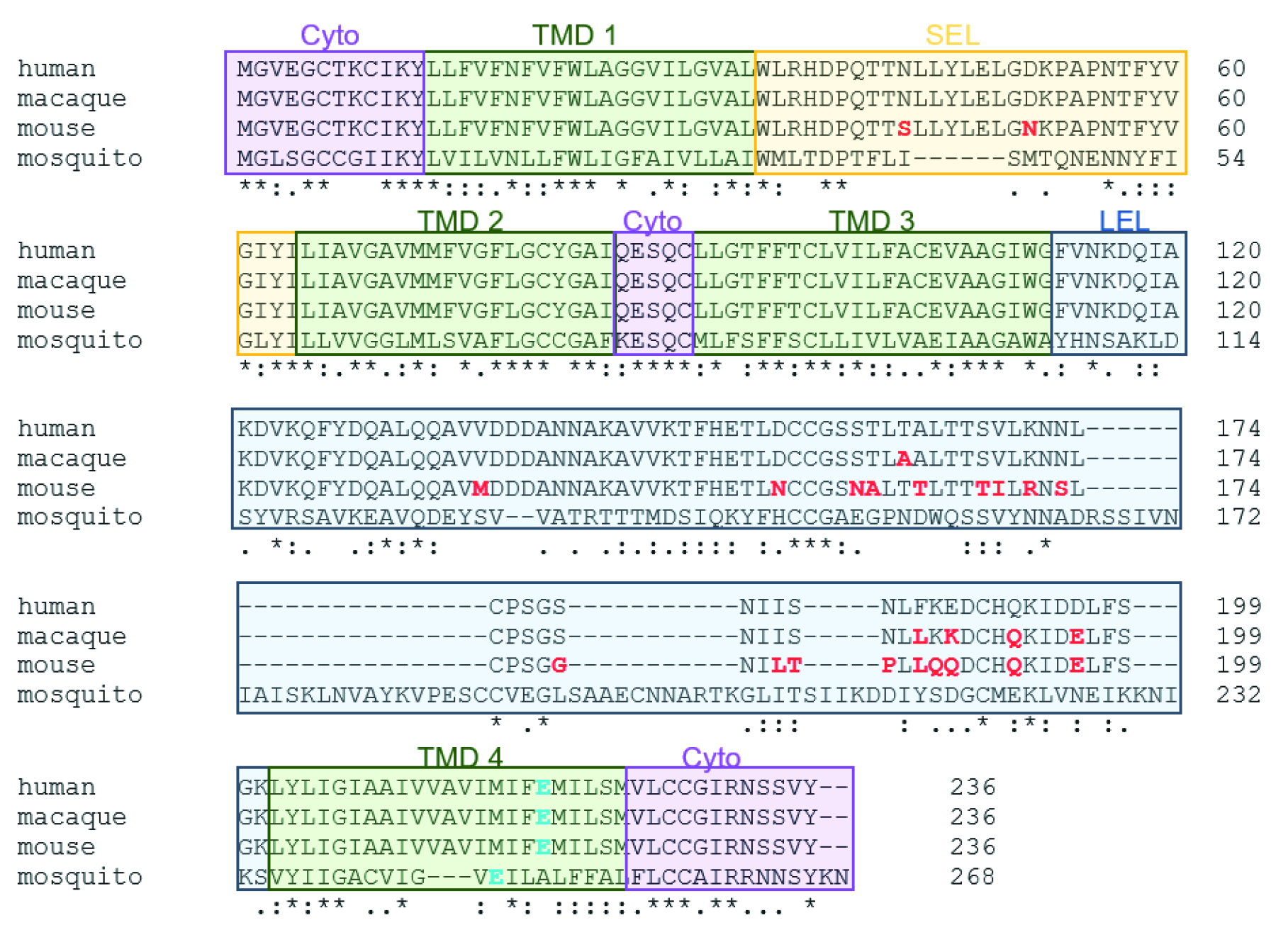

Supplement: FIG S3 [file mbio.00731-22-s0003.tif]
